# Thank you to all of our reviewers in 2020

**DOI:** 10.1038/s42003-021-01719-9

**Published:** 2021-02-19

## Abstract

5314 individual reviewers contributed to the peer review process at *Communications Biology* in 2020. We acknowledge and thank each of them here.

*Communications Biology* received more than 3000 research and review article submissions last year, of which over 1700 were sent for external review. In all, 5314 individuals gave their time to ensure a fair and thorough peer review process in the uniquely challenging year that was 2020. These individuals are recognized below in alphabetical order by surname. We are truly grateful to all of our reviewers. Journal staff take full responsibility for any misspellings, omissions, or other errors.

5314 individuals gave their time to ensure a fair and thorough peer review process in the uniquely challenging year that was 2020.

- Anna M. Åsman
- Adam Abate
- Cory Abate-Shen
- Juliya Abbasi
- Geoffrey Abbott
- Karen Abbott
- Basem Abdallah
- Omar Abdel-Wahab
- Sarki Abdulkadir
- Maha Abdullah
- Ikuro Abe
- Masato Abe
- Ethan Abel
- Oscar Abilez
- Giancarlo Abis
- Jan Pieter Abrahams
- Andrey Abramov
- Jakub Abramson
- Carla Abreu
- Fernanda Abreu
- Gabriel Abril Rodriguez
- Magnus Abrink
- Enas Abu Shah
- Tobias Ackels
- Margaret Ackerman
- Francisco Acosta-Reyes
- Guillermo Acuna
- Oreste Acuto
- Chinnakkaruppan Adaikkan
- Jerzy Adamski
- Bahman Afsari
- Behdad Afzali
- Eneritz Agirre
- Federica Agosta
- Melina Agosto
- Lorena Aguilar-Arnal
- Jose Aguilar-Rodríguez
- Nadia Aguillon-Hernandez
- Golam Ahammed
- Arti Ahluwalia
- Iqbal Ahmad
- Margaret Ahmad
- Munir Ahmad
- Muneeb Ahmed
- Warish Ahmed
- Bumsoo Ahn
- Velma Aho
- Gaurav Ahuja
- Nicole Aiello-Couzo
- Ajoy Ajoy Basak
- Takashi Akagi
- Ali Akbari
- Mohsen Akbari
- Fulya Akcimen
- Seiji Akimoto
- Aleksei Aksimentiev
- David Alabadi
- Monzurul Alam
- Sk. Kayum Alam
- Eric Alani
- Alexandre Alanio
- Gregorio Alanis-Lobato
- Balbino Alarcon
- Nick Albert
- Victor Albert
- Urs Albrecht
- Nathan Alder
- Irina Alecu
- Lauren Aleksunes
- Andy Alexander
- Steven Alexander
- William Alexander
- Andrei Alexandrescu
- Alonzo Alfaro-Núñez
- Nabil-Fareed Alikhan
- Micah Allen
- Peter Allen
- Philip Allen
- Ro Allen
- Scott Allen
- Kristina Allen-Brady
- Prasanna Allu
- Catarina Almeida
- Assaf Alon
- Alenjandra Del Carmen Alonso
- David Alonso
- Hani Alotaibi
- Donát Alpár
- Ahmad Alqudah
- Myriam Altamirano-Bustamante
- Matthias Altmeyer
- Barry Alto
- Douglas Altshuler
- Lucia Altucci
- Fernando Alvarez
- Joao Miguel Alves
- Nuno Alves
- Marina Alves Amaral
- Kaushalya Amarasinghe
- Ivano Amelio
- Chris Amemiya
- Neal Amin
- Shady Amin
- Rogerio Amino
- Shimon Amir
- Khaled Amiri
- Hailong An
- Yu An
- Vaishnavi Ananthanarayanan
- Frank Anderson
- Kevin Anderson
- Richard Anderson
- Rika Anderson
- Anand Andiappan
- Adrian-Ştefan Andrei
- Marco Andrello
- Thomas Andresen
- Ion Andreu
- Andrews Andrew
- Melissa Andrews
- Thomas Andrillon
- Elliot Androphy
- Angelina Angelova
- James Ankrum
- Christina Annunziata
- Tamil Anthonymuthu
- Srdjan Antic
- Michael Antle
- Pierre-Olivier Antoine
- Marc Antoine Sani
- Luiza Aparecido
- Samuel Aparicio
- Sebastien Apcher
- Brian Appleby
- Rajendra Apte
- Catalina Arévalo Ferro
- Maria Aragona
- Mariaceleste Aragona
- Midori Arai
- Kazuharu Arakawa
- Beatrice Aramini
- Beatriz Aranda
- Zolt Arany
- Mehdi Arbabi-Ghahroudi
- Mariona Arbonés
- Nathan Archer
- Alan Archibald
- Jeffrey Ardell
- Alvaro Ardiles
- Mohammad Arif
- Paola Arimondo
- Minetaro Arita
- Dan Arking
- Andrea Armani
- Melissa Armstrong
- Thomas Arnesen
- Roland Arnold
- Nausica Arnoult
- Serge Aron
- Sanjeevani Arora
- Qadeer Arshad
- Steven Artandi
- Yulia Artemenko
- Janelle Arthur
- Simon Arthur
- Ferruh Artunc
- Rajindra Aryal
- Hiroshi Asahara
- Munehiro Asally
- Yasuhisa Asano
- Dominik Aschenbrenner
- Pouyan Asgharzadeh
- Elizabeth Asher
- Fariba Assadi-Porter
- Haruyuki Atomi
- Shota Atsumi
- Bernard Attali
- Karl Attard
- Noam Attias
- Ilaria Attili
- Ina Attrée
- Shannon Wing Ngor Au
- Martina Aubrechtova
- William Auffermann
- Ben Augustine
- Yurii Aulchenko
- Alexander Aulehla
- Tim Aumann
- Didier Aurelle
- William Ausich
- Kellar Autumn
- Mikhail Avdeev
- Bruno Averbeck
- Frank Avila
- Karen Avraham
- Fazli Awan
- Ahidjo Ayouba
- Claus Azzalin
- Klaus Bønnelykke
- Oliver Bäumchen
- Laszlo Bögre
- Bettina Böttcher
- Marco Bürger
- Xiaoliang Ba
- Constance Baaten
- Subash Babu
- Dominik Bach
- Hilmar Badin
- Mirian Baes
- Jared Baeten
- Meisam Bagheri
- Sacha Baginsky
- Michel Baguette
- Sophie Bagur
- Jingbing Bai
- Xiaochen Bai
- Travis Bailey
- Calum Bain
- Emily Baird
- Soren Bak
- Brett Baker
- Chris Baker
- Daniel Baker
- Edward Baker
- Matthew Baker
- Tracy Baker-Herman
- Jeroen Bakkers
- Leonora Balaj
- Cosima Baldari
- Hanna-Mari Baldauf
- Nicholas Balderston
- Megan Baldridge
- Fernando Balestra
- Pedro Ballester
- David Baltrus
- Nigel Bamford
- Laura Banaszynski
- Amitabha Bandyopadhyay
- Dhriti Banerjee
- Manidipa Banerjee
- Duhee Bang
- Marie-Louise Bang
- Anthony Baniaga
- James Bann
- Sebastiano Banni
- Florence Bansept
- Lisui Bao
- Ori Bar-Nur
- Rigmor Baraas
- Tahsin Barakat
- Pavel Baranov
- Joan Barau
- Ivana Barbaric
- Annika Barber
- David Barbie
- Aurel Barbotin
- Thomas Barends
- Thaddeus Bargiello
- Daniel Youssef Bargieri
- Marin Barisic
- Vincenzo Barnaba
- Mike Barnkob
- Celia Baroux
- Blanca Barquera
- Viviana Barra
- Timothy Barraclough
- Rodolphe Barrangou
- Ellen Barrett
- Jeffrey Barrett
- Jessica Barson
- Nicola Barson
- Daniel Barta
- Alexander Bartelt
- Péter Barthó
- Mark Bartlam
- Thomas Bartnikas
- Paolo Bartolomeo
- Dinesh Barupal
- Benjamin Barwick
- Nir Barzilai
- Susan Baserga
- Pouya Bashivan
- Michal Bassani-Sternberg
- Jean-Etienne Bassard
- Holger Bastians
- Anindita Basu
- Avik Basu
- Subhadip Basu
- Brice Bathallier
- Cristina Battaglia
- Alexandra Battaglia-Mayer
- Frederic Batteux
- Fabia Battistuzzi
- Mathilde Baude
- Luc Baudouin
- Joshua Bauer
- Bernhard Baumann
- Christoph Baumann
- Jose Bautista
- Vassiliy Bavro
- Alexis Beaurepaire
- Andrea Becchetti
- Oren Becher
- Melissa Beck
- Sarah Beck
- Benjamin Becker
- Claude Becker
- Damien Becker
- Therese Becker
- Hendrik Beckert
- Stephen Beckett
- Sandra Beer-Hammer
- Luciano Beheregaray
- Petra Beli
- Douglas Bell
- Harold Bell
- Nicholas Bello
- Paola Bellosta
- Uri Ben-David
- Nir Ben-Tal
- Heather Bender
- Valentina Benfenati
- Kingsly Beng
- Itai Benhar
- Yoselin Benitez-Alfonso
- Mehdi Benkahla
- Eric Bennett
- Abby Benninghoff
- Sasisekhar Bennuru
- Joshua Benoit
- Roland Benoit
- Stefania Benonisdottir
- Leslie Berg
- Johannes Berger
- Susanne Berger
- Liza Bergkvist
- Thore Bergman
- Mónica Berjón-Otero
- Ben Berks
- Andreia Bernardo
- Sonja Berndt
- Dirk Bernhardt-Walther
- Jimena Berni
- Annie Bernier
- David Bernlohr
- Matthew Berriman
- Jean-Guy Berrin
- Fred Berry
- Thomas Bertero
- Nicolas Bertho
- Antonio Bertoletti
- Armando Bertone
- George Bertsias
- Alison Bertuch
- Nicolas Bery
- Stephane Besnard
- Robert Best
- Dany Beste
- Sophie Bestley
- Doron Betel
- Charles Bevins
- Jong Bhak
- Kapil Bharti
- Aadra Bhatt
- Arjun Bhattacharya
- Somanon Bhattacharya
- Sudeepa Bhattacharyya
- Subrat Bhattamisra
- David Bhella
- Yoshita Bhide
- Huichang Bi
- Piero Bianco
- Jennifer Biddle
- Anna Bielak-Zmijewska
- Martina Bielaszewska
- Jan Bieschke
- Kasia Bieszczad
- Monika Bijata
- Else Bijker
- Janine Bijsterbosch
- Moses Bility
- Marc Bilodeau
- George Birchenough
- James Birchler
- Jeremy Bird
- Einar Birkeland
- Marc Birtwistle
- Jordan Bisanz
- Anthony Bishop
- Alexandre Bisson
- Gal Bitan
- Moshe Biton
- Kyle Bittinger
- Mariano Bizzarri
- Joshua Black
- Benjamin Blackman
- Julianna Blagih
- David Blair
- Stephen Blake
- Antonio Blanco
- Francisca Blanco Herrera
- Philippe Blancou
- Robert Blankenship
- Maria Blasco
- Bradley Blaser
- Janusz Blasiak
- Mark Blaskovich
- Cornelis Blauwendraat
- Petra Bleeker
- Ran Blekhman
- Timothy Blenkinsop
- Paul Blischak
- Nicholas Block
- Dieter Blottner
- Christian Blum
- Ruben Boado
- Amy Boal
- Agata Bochynska
- Andreas Bock
- Anja Bockmann
- Örjan Bodin
- Ines Boehm
- Ralph Boettcher
- Tiffany Bogich
- Jörg Bohlmann
- Lukasz Bola
- Alessandra Boletta
- Aureliano Bombarely
- Joe Bondy-Denomy
- Susan Bonner-Weir
- Titouan Bonnot
- Geert-Jan Boons
- Stephanie Booth
- Selina Bopp
- Alisdair Boraston
- Rebecca Borchering
- Francois Bordeleau
- Patricia Bordes
- Angelique Bordey
- Adair Borges
- Nicolas Borisov
- Tatiana Borisova
- Maria Borisova-Mubarakshina
- Thomas Bornschlögl
- Alexander Borodavka
- David Borton
- Jeffrey Bose
- Peter Bossaerts
- João Botelho
- Hector Botella
- Jennifer Botha
- Francesca Bottanelli
- Emmanuel Boucrot
- Eric Bouhassira
- Jean-Baptiste Boulé
- Travis Bourret
- Kristen Bouska
- Vassiliki Boussiotis
- Sophie Bouton
- Benjamin Boutrel
- Cara Boutte
- Christina Boyle
- Yuri Bozzi
- Rogier Braakman
- Ryan Bracewell
- Frank Bradke
- Sven Bradler
- Daniel Bradley
- Ulrike Braeckman
- Mattia Bramini
- Pedro Branco
- Giovanni Brandi
- Sebastian Brauchi
- Alejandra Bravo
- Tinatin Brelidze
- Juliane Bremer
- Cormack Brendan
- Christophe Bressac
- Sophie Breton
- Damien Brevers
- Gregory Brewer
- Ester Brielle
- Roberta Brinton
- Marcela Brissova
- Anne Britt
- Philip Britz-Mckibbin
- William Broadhurst
- Mathieu Brochet
- Susan Brockerhoff
- Petter Brodin
- Els Broens
- Stephen Brohawn
- Johannes Broichhagen
- Paul Brookes
- Jeffrey Brooks
- Sébastien Brosse
- Marco Brotto
- Grant Broussard
- Grant Brown
- James Brown
- Susan Brown
- Jeffrey Browning
- Patricia Brubaker
- Spencer Brucks
- Emily Brudner
- Thomas Brueser
- Pierre Bruhns
- Caroline Brun
- Alejandra Bruna
- Frédéric Brunet
- John Bruning
- Corina Brussaard
- Donald Bryant
- Mark Brynildsen
- Ola Brynildsrud
- Robert Bryson-Richardson
- Lijing Bu
- Wen Bu
- Gregor Bucher
- Anthony Buckley
- Ann Bucklin
- Kurtis Budden
- Matthew Buechler
- Paul Buehler
- Cliff Bueno de Mesquita
- Gisela Buetner
- Rochelle Buffenstein
- Eric Buffetaut
- Katrine Bugge
- Stéphane Buhler
- Anwen Bullen
- George Bullerjahn
- Alex Bullock
- Serdar Bulun
- Christopher Buneo
- Aaron Burberry
- Christoph Burckhardt
- Kathryn Burdon
- Pamela Burger
- Elizabeth Burgess
- Graham Burgess
- Stephen Burgess
- Nicola Burgess-Brown
- John Burke
- Oliver Burren
- Christopher Burridge
- Florence Burté
- Adam Burton
- Marc Aurel Busche
- Julia Busik
- Matthew Butchbach
- Roger Butlin
- Elisabeth Butz
- Mark Byrne
- Matthew Byrne
- Patrick Byrne
- James Byrnes
- Adam Byron
- Danilo Bzdok
- Carlos Cáceres
- Stephanie Cabantous
- Filipe Cabreiro
- Edwin Cadena
- Stefano Cagnin
- Valeria Cagno
- Lanlan Cai
- Liang Cai
- Ling Cai
- Xiujun Cai
- Yunpeng Cai
- J. Mauro Calabrese
- Joaquín Calatayud
- David Calderwood
- Heather Caldwell
- Rocco Caliandro
- Andrea Calixto
- Brian Callahan
- Fabiano Calmasini
- Eva María Camacho
- Alexander Cameron
- Stefano Campanaro
- Katie Campbell
- Chiara Campiglio
- Brice Campo
- Javier Campos-Gomez
- Alberto Canarini
- Carlos Canchaya
- Michael Cangkrama
- Nuria Canibe
- Aurore Canoville
- Cinzia Cantacessi
- Laura Cantini
- Harvey Cantor
- Margherita Cantorna
- Hongnan Cao
- Song Cao
- Clemente Capasso
- Pol Capdevila
- J. Gregory Caporaso
- Sara Capponi
- Emidio Capriotti
- Simona Carbone
- Pablo Carbonell
- Rebecca Cardigan
- Silvia Cardona
- Stephana Carelli
- Daniel Carlson
- Jeremy Carlton
- Santiago Carmona
- David Caron
- Sebastien Carpentier
- Guido Carpino
- Serena Carra
- Nestor Carrillo
- Jimena Carrillo-Tripp
- Victor Carrion
- Marc Carriquí
- Stephanie Carroll
- Rita Carsetti
- Ana Luisa Carvalho
- Michael Cashel
- Charles Caskey
- Tamara Caspary
- Corinne Cassier-Chauvat
- Carlos Castañeda
- Natalia Castano-Rodriguez
- Pau Castel
- Immacolata Castellano
- Filipe Castro
- Rosemary Cater
- James Cavanagh
- Simone Ceccobelli
- Germano Cecere
- Thomas Cech
- Francesca Ceroni
- Sarah Certel
- Vincenzo Cerullo
- Camilla Cerutti
- Fabrizia Cesca
- Barbaros Cetin
- Oliver Chén
- Young Cha
- Jenu Chacko
- Samuel Chaffron
- Chrispin Chaguza
- Solomon Chak
- Debopam Chakrabarti
- Joy Chakraborty
- M. Mallar Chakravarty
- Janice Chambers
- Joe Chambers
- Patrick Chames
- Cheong Xin Chan
- Clement Chan
- Cliburn Chan
- David Chan
- Eva Chan
- Jo-Anne Chan
- Stephen Chan
- Wai Kit Chan
- Natali Chanaday
- Navdeep Chandel
- Josephine Chandler
- Talon Chandler
- Anchal Chandra
- Uma Chandrachud
- Mahesh Chandrasekharan
- Edward Chang
- Eric Chang
- Matthew Chang
- Nan-Shan Chang
- He Changjiu
- John Chaput
- Yee-Yung Charng
- Elena Chartoff
- Emily Chase
- Qasim Chaudhry
- Dipayan Chaudhuri
- Indrajit Chaudhury
- Radha Chauhan
- Damien Chaussabel
- Sylvain Chauvette
- Philippe Chavrier
- Umber Cheema
- Albert Chen
- Anping Chen
- Baoshan Chen
- Benjamin Chen
- Caiyan Chen
- Chi-Hua Chen
- Chien-Fu Chen
- Chongyi Chen
- Chun-Chi Chen
- Chunli Chen
- Dijun Chen
- Feng Chen
- Gefei Chen
- Guo-Qiang Chen
- Haiyang Chen
- Hangzi Chen
- Jian Chen
- Jian-Feng Chen
- Jianhan Chen
- Jichao Chen
- Jie Chen
- Junjie Chen
- Kong Chen
- Leilei Chen
- Liang Chen
- Peilin Chen
- Xiao-Lin Chen
- Xin Chen
- Xue-Xin Chen
- Xuechu Chen
- Yan Chen
- Yanan Chen
- Yaodong Chen
- Ye-Guang Chen
- Yujie Chen
- Yupeng Chen
- Zhe Chen
- Zhong Chen
- C. Yan Cheng
- Daojun Cheng
- Feixiong Cheng
- Keith Cheng
- Tingcai Cheng
- Wei Cheng
- Yifan Cheng
- Yong Cheng
- Simona Chera
- Peter Cherepanov
- Andrew Cherniack
- Douglas Chesters
- Jonah Cheung
- Fabienne Chevance
- Germain Chevignon
- Jeannie Chew
- Chuan Chiang-Ni
- Eduardo Chini
- Christine Chio
- Stephen Chiswell
- Kenny Chitcholtan
- Dong-Hyung Cho
- Hyeseong Cho
- Raymond Cho
- Sohyun Cho
- Sung-Joon Cho
- James Chodosh
- Regine Choe
- Hee-Jung Choi
- Hélène Choquet
- Edward Kai-Hua Chow
- Eric Chow
- Louise Chow
- Seong Hoong Chow
- Antonios Christou
- Kendra Chritz
- Kai-Hsiang Chuang
- Sung Chun
- Chun-Wa Chung
- Eun Ji Chung
- Hee Jung Chung
- John Chute
- Lily Chylek
- George Cicila
- Lena Ciric
- Carmen Cirstea
- Francesca Citron
- Danielle Claar
- Mareli Claassen
- Mireille A. Claessens
- Emily Clark
- Ingra Claro
- Alison Cleary
- Timothy Cleland
- Thomas Clements
- Gemma Clucas
- David Clynes
- Martin Clynes
- Aurelie Cobat
- Franca Codazzi
- Enrico Coen
- Daniel Coffey
- Rodrigo Cofre
- Michael Cohen
- Ivan Colaluca
- Jacques Colinge
- Jacques-Philippe Colletier
- Karen Colley
- Claudio Collinet
- Joanna Collingwood
- Allen Collins
- Helen Collins
- Rebecca Colman
- Giorgio Colombo
- Lucy Colwell
- Karen Colwill
- Lorenza Colzato
- Erika Comasco
- Simon Comerma
- Mariana Conceicao
- Carlo Condello
- Marc Connor
- Chris Conroy
- Christos Constantinidis
- Pio Conti
- Jose Contreras-Vidal
- Samantha Cook
- Ira Cooke
- Marion Coolen
- Jan Cools
- Janine Coombes
- Margery Coombs
- Robin Cooper
- Cyril Corbet
- Vincenzo Corbo
- Julia Cordero
- Cinzia Corinaldesi
- Peter Corkeron
- Omar Cornejo
- Dawn Cornelison
- Robert Cornell
- Luc Cornet
- Evan Cornett
- Gianluca Corno
- Benjamin Corona
- Miguel Corona
- Nicolas Corradi
- Rebecca Corrigan
- David Cortez
- Kristina Corthals
- Alexandre Corthay
- Francesca Coscia
- Stephen Cose
- Ahmet Coskun
- Luis Costa
- Paola Costelli
- Mark Costello
- Blair Costelloe
- Susan Cotman
- Chris Cotsapas
- John Couchman
- Cédric Coulouarn
- Pascal Coumailleau
- Laurent Counillon
- Gregoire Courtine
- Bruno Coutard
- James Cowan
- Peter Cowman
- Georgina Cox
- Philip Cox
- Peter Crack
- Erin Cram
- Robert Cramer
- Rolf Craven
- Meaghan Creed
- Valérie Crepel
- Simona Cristea
- Elizabeth Crofton
- Terence Crofts
- John Cronan
- Justin Cross
- Michael Crossley
- Michael Douglas Crowther
- Perrine Cruaud
- Felix Cruz
- Luis Cuello
- Bianxiao Cui
- Yiping Cui
- Edna Cukierman
- Richard Culleton
- Alexander Culley
- Thiago Cunha
- Brian Cunningham
- Chris Cunningham
- John Cunningham
- Kathryn Cunningham
- Hermann Cuntz
- Tom Cupedo
- Tyler Curiel
- Geoff Currie
- Emilio Cusanelli
- Marija Cvetanovic
- Lukasz Cwiklik
- Michael Cynamon
- Michael Czubryt
- Marcos Díaz-Gay
- Fabrizio D'Adda Di Fagagna
- Maurizio D'Antonio
- Luigi D'Ascenzo
- Michael D'Emic
- Kauê da Costa
- Fernando da Silva
- Gabriela da Silva Xavier
- Marcel Daadi
- Daniele Daffonchio
- Rafael Daga
- Onur Dagliyan
- Lina Dagnino
- Gerhard Dahl
- Lisheng Dai
- Alma Dal Co
- Athanasios Dalakouras
- Zachary Dalebroux
- Kevin Daly
- Paola Dama
- Lars Riis Damgaard
- Adam Damry
- Ajai Dandekar
- Farhad Danesh
- Emily Dangremond
- Carolin Daniel
- Michael Dannemann
- Davide Danovi
- Gautam Dantas
- Tiago Dantas
- Altaf Dar
- Fabien Darfeuille
- Frederic Darios
- Hugo Darras
- Chandrima Das
- Pran Datta
- Sandeep Robert Datta
- Thomas Daubon
- Gary Daughdrill
- Katie Daughters
- Oliver Daumke
- Fabian Davamani
- Yael David
- Lance Davidson
- Christopher Davies
- John Davies
- Carla Davis
- Ryan Davis
- Scott Davis
- Michelle Dawson
- Sally Dawson
- Chi-Ping Day
- David Day
- Kenneth de Baets
- Marta de Barba
- Pietro de Camilli
- Guillermo de Carcer
- Daniel de Carvalho
- Geoffrey de Couto
- Johan-Owen de Craene
- Joice de Faria Poloni
- Marcus de Goffau
- Bert Dde Groot
- Vinicio de Jesus Perez
- A.P. Jason de Koning
- Tania de Koning-Ward
- César de la Fuénte
- Eva de Lago
- Primal de Lanerolle
- Peter de Lange
- Guillaume de Lartigue
- Irene de Lazaro
- Raphael de Lira
- Victor de Lorenzo
- Paolo de Los Rios
- Daniele de Luca
- Alex De Marco
- Ramon Andrade de Mello
- Alex de Mendoza
- Fernando de Miguel
- Noel de Miranda
- Nicole de Nisco
- Erik de Schutter
- Paulo de Souza
- Fabrizio de Vico Fallani
- Maarten de Vos
- Franciska de Vries
- Frans de Waal
- Marcel de Zoete
- Greg Deangelis
- Margaret Deangelis
- Galia Debelouchina
- Charles Deber
- T. Alexander Dececchi
- Anabelle Decottignies
- Franck Dedeine
- Joris Deelen
- Bridget Deemer
- James Degregori
- Scott Dehm
- Alexander Deiters
- Borislav Dejanovic
- Giannino Del Sal
- Marc Delarue
- Luis Delaye
- Michela Deleidi
- Lucie Delemotte
- Cédric Delevoye
- Mauricio Delgado
- Brian Delisle
- Jerome Delroisse
- Tonya Delsonto
- Neal Deluca
- Emmanuel Demont
- Han-Xiang Deng
- Ruijie Deng
- Tao Deng
- Xianding Deng
- Thomas Denk
- Daniel Denman
- Eileen Denovan-Wright
- Paul Denton
- Robert Denver
- Martin Denzel
- Wannes Dermauw
- Priya Deshpande
- Michèle Desjardins
- Anne Deslattes Mays
- Paula Desplats
- Philippe Després
- Annie Desrochers
- Carmen Dessauer
- Claire Detrain
- Jörg Deutzmann
- Christian Devaux
- Lakshmi Devi
- Gautam Dey
- Avantika Dhabaria
- Guha Dharmarajan
- Sirano Dhe-Paganon
- Pal Dhiman
- Xin Di
- Ferdinando Di Cunto
- Simone Di Giovanni
- Francesco Saverio Di Leva
- Lucia Di Marcotullio
- Paola Di Meglio
- Francesca Di Modugno
- Annalisa Di Ruscio
- Elia Di Schiavi
- Jean-Simon Diallo
- Fernando Diaz
- Mani Diba
- Angela Dibenedetto
- Matthew Dicks
- Susanne Diekelmann
- Seydina Diene
- Herman Dierick
- Sarah Diermeier
- Ilka Diester
- Dennis Dietzen
- Ronald Dijkman
- Paul Dijkstra
- Panayiotis Dimitrakopoulos
- Stavros Dimitriadis
- Dimiter Stanchev Dimitrov
- Rumiana Dimova
- Astra Dinculescu
- Baoqing Ding
- Huangen Ding
- Qiurong Ding
- Xiaoyun Ding
- Ye Ding
- Yuchen Ding
- Ottavia Dipasquale
- Luisa Ann Dipietro
- Thomas Dittmar
- Alexander Dityatev
- Ajit Divakurani
- Rose Dixon
- Alexandre Djiane
- Kristina Djinovic-Carugo
- Ana Djukovic
- Jorge Doña
- Carlota Dobaño
- Ian Dobbins
- Ross Dobie
- Chris Dockendorff
- Antony Dodd
- Greg Dodge
- James Dodge
- Karen Doggett
- Priyanka Dogra
- Tatsuya Dokoshi
- Ylli Doksani
- Jason Doles
- Vernon Dolinsky
- Anna Doloman
- Helena Sofia Domingues
- Javier Domínguez Zamorra
- Stefano Donadio
- Janet Donaldson
- Justin Donato
- Jing Dong
- Tien Dong
- Yan Dong
- Emma Dorris
- David Dougan
- Stephanie Dougan
- Pamela Douglas
- Steven Dowdy
- Jennifer Dowling
- Timothy Downing
- Andreas Dräger
- Gang Changcheng
- Olga Dragoy
- Alexander Drakesmith
- Marlène Dreux
- David Drew
- Kelly Drew
- Matthias Drosten
- Jianhai Du
- Jianzhong Du
- Wei Du
- Liting Duan
- Xiaojie Duan
- Tiago Duarte
- Grzegorz Dubin
- Jessica Dubois
- Marcus Duchler
- Jorge Duconge
- Agnieszka Dudkiewicz
- Giles Duffield
- Yves Dufrene
- Priya Duggal
- Iain Duggin
- Thomas Duhen
- Reuven Dukas
- Guillaume Dumas
- Alexander Dunhill
- Jessica Dunleavy
- Mary Dunlop
- Luke Dunning
- Jared Alexander Dunnmon
- Sirio Dupont
- Julian Dupuis
- Beatrice Durand
- Leonardo Durante
- Andrés Durantini
- Walter Durka
- Ranjan Dutta
- Marie-Cécilia Duvernoy
- Birge Özel Duygan
- Zdenek Dvorak
- Hany Dweck
- Roisin Dwyer
- Michael Dyer
- Marcus Dymond
- Lars Dyrskjot Andersen
- Elaine Dzierzak
- Barbora East
- Alice Easton
- Philip Eaton
- Jaelyn Eberle
- Jeff Eble
- Mo Ebrahimkhani
- Melanie Eckersley-Maslin
- Anastassios Economou
- Chandrakanth Edamakanti
- Thomas Edwards
- Jorine Eeftens
- Robert Eferl
- Rouslan Efremov
- Mirjana Efremova
- Martin Egeland
- Dan Ehninger
- Gregory Ehx
- Jutta Eichler
- Brandt Eichman
- Judith Eisen
- Raphael Eisenhofer
- Karin Eisinger
- Nady El Hajj
- Wael El-Nachef
- Sam El-Osta
- Yasmine El-Shamayleh
- Satheesh Elangovan
- Michael Elbaum
- Ihab Eldessouki
- Kevin Elias
- Fredrik Elinder
- Eyad Elkord
- Philip Elks
- Christopher Elliott
- Kerryn Elliott
- Mark Ellisman
- Courtney Ellison
- Wilfried Ellmeier
- Arne Elofsson
- Maija-Leena Eloranta
- Alberto Elosegui-Artola
- Wael Elshamy
- Joanne Emerson
- Ben Emery
- Melissa Emery Thompson
- Francisco Encinas-Viso
- Toshiya Endo
- Lauren Endres
- Jutta Engel
- Christine Engeland
- Christoph Englert
- Martin Engqvist
- Luis Enjuanes
- Daniel Enosi Tuipulotu
- Tomas Erban
- Marc Erhardt
- Aaron Ericsson
- Jeffrey Erlich
- Joseph Erlichman
- Maria Ermolaeva
- Jason Ernst
- Lynda Erskine
- Dani Eshel
- Mark Esposito
- Borja Esteve-Altava
- Ana María Estrada-Sánchez
- Kayvan Etebari
- Iryna Ethell
- Anders Etzerodt
- Manuel Etzkorn
- Frank Etzler
- Jaime Eugenin
- Julian Evans
- Louise Evans
- Tom Evans
- Artem Evdokimov
- Damien Eveillard
- Kathryn Everson
- Denis Evseenko
- Alice Exnerová
- Patrick Eyers
- Teresa Ezponda Itoiz
- Ellen Ezratty
- Reinhard Fässler
- Boris Fürtig
- Luca Fabris
- Abigail Fahim
- J. Tyler Faith
- Bryce Falk
- Joseph Falke
- Peter Falkingham
- Peter Faller
- Pascal Falter-Braun
- Jean Fan
- Kelong Fan
- Qiao Fan
- Chen-Jie Fang
- Ning Fang
- Christian Fankhauser
- Michael Fanselow
- Katherine Fantauzzo
- Dominic Fareri
- Hesso Farhan
- Michael Farkas
- Evan Farkash
- William Farnaby
- Per Fauchald
- Christine Faulkner
- David Favero
- Peter Fecci
- Vadim Fedorov
- Alan Feduccia
- Pawel Fedurek
- Anthony Fehr
- Michael Feigin
- Larry Feinstein
- Brunie Felding
- L.F. Felicori
- Gidon Felsen
- Sebastien Felt
- Markus Fendt
- Baomin Feng
- Liang Feng
- Qili Feng
- John Fennell
- Leif Fenno
- Scott Ferguson
- Shawn Ferguson
- Ana Fernández
- Juan Fernández-Recio
- Ricardo Fernandes
- Luis Angel Fernandez
- María de la Paz Fernández
- Soledad Fernandez
- Llorenc Fernandez-Coll
- Erika Fernandez-Vizarra
- Juan Ferré
- Mariella Ferrante
- Fortunato Ferrara
- Joao Ferreira
- Jorge Ferreira
- Manuel Ferrer
- Laura Ferrer-Font
- David Ferrier
- Christine Ferrier Pages
- James Ferry
- Hendrik Feys
- Richard Ffrench-Constant
- Gentile Francesco Ficetola
- Jakub Piotr Fichna
- Andrew Fielding
- Jens Fielitz
- Martijn Figee
- Karen Filbee-Dexter
- Massimo Filippi
- Geoffrey Findlay
- Helen Findlay
- Andres Finzi
- Alessandro Fiorenzano
- Josh Firth
- M. Dominik Fischer
- Carola Fischer-Tenhagen
- Charles Fisher
- Jenny Fisher
- Harold Fisk
- Robert Fitak
- Slyvia Fitting
- Una Fitzgerald
- Teresa Fitzpatrick
- Jason Flannick
- Holger Flechsig
- Melanie Flint
- Hauke Flores
- Mario Flores
- Dorothea Floris
- Patrick Floris
- John Foerster
- Sylvain Foissac
- Edward Fon
- Loren Fong
- Vera Fonseca
- Gustavo Fontecha
- Lorenzo Fontolan
- Stuart Forbes
- Natalie Forde
- Stephanie Forkel
- Stefan Forster
- Patrice Fort
- Mikael Fortelius
- Patrick Forterre
- Daniel Foster
- Leonard Foster
- Timothy Foster
- William Foster
- Karim Fouad
- Fabienne Foufelle
- Chris Foulon
- Stephanie Fowler
- Kasey Fowler-Finn
- Stefan Frässle
- Karine Frénal
- Jörg Fröbisch
- Marco França
- Nora Franceschini
- Anna Francesconi
- Christopher Francklyn
- Michelle Francl
- Ricardo Franco
- Rodrigo Franco
- Achilleas Frangakis
- Philipp Franken
- Susanne Franssen
- Laurent Frantz
- Titus Franzmann
- Wayne Frasch
- Danielle Fraser
- Karl Fraser
- Marie Fraser
- Katrin Frauenknecht
- Brian Fredensborg
- Daniela Freitas
- Alexander Fremier
- Andrew French
- Christian Frezza
- W. Florian Fricke
- Simon Fricker
- Steven Friedenberg
- Peter Friedl
- Daniel Friedrich
- Seth Frietze
- Daniel Frigo
- Jiri Friml
- Stephen Frings
- Renato Frischknecht
- Sabrina Fritah
- Marco Fritzsche
- Marcus Frohme
- Bastian Fromm
- Raimund Fromme
- Dominique Frueh
- Dan Fu
- Tian-Ming Fu
- Xianghui Fu
- Xinmiao Fu
- Yang-Xin Fu
- Yaron Fuchs
- Gustavo Fuertes
- Haruhiko Fujiwara
- Akiyoshi Fukamizu
- Tomokazu Fukuda
- Ben Fulcher
- James Fuller
- Patrick Fuller
- Thomas Fung
- Yoshiaki Furukawa
- Francisco Gámez
- Gustavo Gámez
- Karina Gómez
- Claudia Göttsch
- Babett Günther
- Alexander Gabibov
- Yannick Gachet
- Parag Gad
- Suresh Gadde
- Theodorus Gadella
- Maciej Gagat
- Steffen Gais
- Maria Gaiser
- Juan Carlos Galán
- Alexander Galkin
- David Gallacher
- Jennifer Gallagher
- Peter Gallant
- Irene Gallego
- Courtney Gallen
- Thierry Galli
- Jenna Gallie
- Jennifer Gallop
- Imed-Eddine Gallouzi
- Andrea Galmozzi
- Iván Galván-Femenía
- Vivian Gama
- Benoit Gamain
- Alicia Gamboa-Debuena
- Noor Gammoh
- Ziv Gan-Or
- Massimo Ganassi
- Karuna Ganesh
- Sreeramaiah Gangappa
- Karunesh Ganguly
- Caixia Gao
- Haichun Gao
- Xinghua Gao
- Yong-Gui Gao
- Gyozo Garab
- Christoph Garbers
- Gabriela García-Guzmán
- María García-Pedrajas
- Francisco García-Rosales
- Claudio Araya Garcia
- Melissa Garcia
- Pablo Garcia Palacios
- Alba Garcia-Lino
- David Gardner
- George Garinis
- Jennifer Garrison
- Fabio Gasparini
- Natalie Gass
- Susan Gasser
- Robert Gatenby
- Christos Gatsogiannis
- Michael Gatza
- Allison Gaudinier
- Arnaud Gautier
- Leonid Gavrilov
- Yubin Ge
- Teklab Gebregiworgis
- Paul Geeleher
- Scott Geib
- James Geiger
- Edward Geisinger
- Werner Geldenhuys
- Mauro Gemmi
- Maria Genander
- Louis Gendron
- Eulalia Genesca
- Yong-Jian Geng
- Guy Genin
- Benoit Gentil
- George Gentsch
- Paul George
- George Georgiou
- Juliana Geraldo
- Anthony Gerber
- Gabriele Gerlach
- Malte Gersch
- Moritz Gerstung
- Jason Gertz
- Kristina Gervin
- Klaus Gerwert
- Stephanie Getz
- Andrew Gewirtz
- Arindam Ghatak
- Reena Ghildyal
- Fabrizio Ghiselli
- Arkasubhra Ghosh
- Pushpita Ghosh
- Rita Ghosh
- Sayan Ghosh
- Michael Giacomelli
- Evangelos Giamarellos‐Bourboulis
- Georgios Giamas
- Maria Giavazzi
- Ted Gibson
- Carmela Giglione
- Jose Pedro Gil
- Clement Gilbert
- Luke Gilbert
- Pavlo Gilchuk
- Tristan Gilet
- Jacquelyn Gill
- Todd Gilligan
- Thomas Gillingwater
- Ana Giménez-Arnau
- Fiona Ginty
- Valentina Giorgio
- Gabrielle Girardeau
- Sylvain Giroud
- Christopher Gisriel
- Daniel Gitler
- Douglas Gladue
- David Glanzman
- Michelle Glass
- Elizabeth Glater
- Sarah Glaven
- Lani Gleason
- Mary Gleason
- Martijn Gloerich
- Mark Glover
- Alexey Glukhov
- Robert Gniadecki
- Geoffrey Gobert
- Susana Godinho
- Chandraiah Godugu
- John Godwin
- Joanna Godzien
- Deniz Goekbuget
- Ramesh Goel
- Marlos Goes
- Laura Goetz
- Rajan Gogna
- Nadine Gogolla
- Boon Cher Goh
- Michel Gohar
- Ethan Goldberg
- Michael Goldberg
- Alexander Golden
- Sam Golden
- Aaron Goldman
- Kezia Goldmann
- Cynthia Goldsmith
- Sandra Gollnick
- Bernard Gomez
- Fabian Gomez
- Damià Gomila
- Roger Gomis
- Raquel González-Fernández
- Kevin Andrew Gonzales
- Susana Gonzalo
- Hani Goodarzi
- David Goode
- John Goodier
- David Goodrich
- Vishrawas Gopalakrishnan
- Vera Gorbunova
- Dmitry Gordenin
- David Gordon
- Emma Gordon
- Reuven Gordon
- Rupa Gordon
- Julia Gorelik
- Kevin Gori
- Fabien Gosselet
- Cara Gottardi
- Nicole Gottdenker
- Roberta Gottlieb
- Magdalena Gotz
- Bruno Goud
- Alexandros Goulas
- Marie José Goumans
- Eleni Gourgou
- Velu Govindan
- Elena Govorunova
- Humaira Gowher
- Aymeric Goyer
- David Goyer
- Jesse Goyette
- Peter Gräber
- Gerhard Grüber
- Thomas Grünewald
- Fabian Grabenhorst
- Anna Grabowska
- Magda Grabowska
- Gregory Grabowski
- Elena Gracheva
- Agnieszka Graczyk-Jarzynka
- Rachel Graham
- Stewart Graham
- W. Vallen Graham
- Marc Graille
- Anthony Gramolini
- Elisa Granato
- Joanes Grandjean
- James Grau
- Erin Greaves
- Alex Green
- Rebecca Green
- Robert Greenberg
- Michael Greenfield
- Alissa Greenwald
- Elisa Greggio
- Veronica Gregis
- Alexander Gregorieff
- Christopher Gregory
- Victor Greiff
- Anna Greka
- Parbir Grewal
- Shiv Grewal
- Arjan Griffioen
- Nicolas Grillet
- Daniel Grimanelli
- Jan Grimm
- Jeff Grimm
- Natasha Grimsey
- Valery Grinevich
- Francesca Grisoni
- Sergei Grivennikov
- Kay Grobe
- Michael Groll
- Alecia Gross
- David Gross
- Robert Grosse
- Steven Grossman
- David Grossnickle
- Madhusudan Grover
- Edward Grow
- Matthew Grubb
- Michael Grubb
- Dean Grubbs
- Zorana Grubic
- Kristina Gruden
- Benjamin Gruenbaum
- Marcus Grueschow
- Luke Grundy
- Berkley Gryder
- Jian Gu
- Le Luo Guan
- Qingdong Guan
- Michael Guarnieri
- Andrea Gubas
- Scott Guelcher
- Michael Guertin
- Michele Guescini
- Borhane Guezguez
- Jeremy Guggenheim
- Orane Guillaume-Gentil
- Karen Guillemin
- Marina Guillen
- Guillaume Guinot
- Tanuj Gulati
- Li Guo
- Ming-Lei Guo
- Tai Guo
- Wei Guo
- Wenjun Guo
- Xiaonan Guo
- Yanjun Guo
- Krishan Gupta
- Rani Gupta
- Shiri Gur-Cohen
- Martin Gustavsson
- Arvid Guterstam
- Julian Gutt
- Vishwesha Guttal
- Valeria Guzman-Luna
- Peter Gyarmati
- Ulf Gyllensten
- Martin Häring
- Terence Hébert
- Christer Höög
- Martin Högbom
- Julia Höglund
- Andi Haas
- Arthur Haas
- Astrid Haase
- Maher Abou Hachem
- David Hackos
- Lauren Hadley
- Ziad Hafed
- Michael Haffner
- Peter-Leon Hagedoorn
- Ji-Sook Hahn
- Nasir Haider
- Sarah Haigh
- Niina Haiminen
- Taiki Hakozaki
- Christopher Halbrook
- Georg Halder
- Tracy Hale
- Chris Haley
- Barbara Halkier
- Susannah Hallal
- Stefan Hallermann
- Michelle Halls
- Elizabeth Hambleton
- Peter Hamilton
- Edith Hammer
- Almuth Hammerbacher
- Michael Hammerling
- Ulrich Hammerling
- Gerald Hammond
- Nils Hampe
- Aidong Han
- Gang Han
- Hu Han
- Leng Han
- Li-Li Han
- Summer Han
- Sung Ok Han
- Xikun Han
- Xue Han
- Yi Han
- Abdulsamie Hanano
- Mary Ann Handel
- Steven Hanes
- Balazs Hangya
- Bertrand Hankoua
- Roger Hanlon
- Franziska Hanschen
- Christian Hansel
- Carsten Hansen
- Kasper Hansen
- Paul Hansen
- Oliver Hantschel
- Nanjing Hao
- Shane Harding
- Pierre Hardy
- Jonathan Hare
- Joshua Hare
- Karim Harhouri
- Arif Harmanci
- Nathaniel Harnett
- Lynsey Harper
- Ed Harris
- Patrick Harris
- Robert Harris
- David Harrison
- Gerald Hart
- Tom Hart
- Kaisa Hartikainen
- Christine Hartmann
- Erica Hartmann
- Anika Hartz
- H. Criss Hartzell
- Ben Harvey
- Robert Harvey
- David Haselbach
- Uri Hasson
- Yutaka Hata
- Keiichi Hatakeyama
- Nathaniel Hathaway
- Motoyuki Hattori
- Yukako Hattori
- Vasili Hauryliuk
- Lars Hausfeld
- Robert Hausinger
- Cheryl Hawkes
- Nicole Hawkins
- Stephen Hawkins
- Dror Hawlena
- Jesse Hay
- Kanako Hayashi
- Yohei Hayashi
- Lee Haynes
- Yuan He
- Luke Healy
- Luke Hearne
- Martin Hebart
- Nikolai Hecker
- Rainer Hedrich
- Lizbeth Hedstrom
- Sarah Hedtrich
- Kavita Hegde
- Johann Heider
- Christine Heilmann
- Ayelet Heimowitz
- Nadine Hein
- Frank Heinrich
- Anna Heintz-Buschart
- Peter Heintzman
- Carl-Philipp Heisenberg
- Christian Heiss
- Ellen Heitzer
- James Hejtmancik
- Sophie Helaine
- Christoph Held
- Marcus Heldmann
- Randolph Helfrich
- Åslaug Helland
- Martin Helmkampf
- Emily Hemann
- Calvin Henard
- William Hendricks
- Matthias Hennig
- Simon Hennig
- Rob Henning
- Robert Henry
- Jonathan Henshaw
- Michael Henshaw
- Richard Henson
- Chelsea Hepler
- Yann Herault
- Guillaume Herbet
- Timothy Hercus
- Patrick Herendeen
- Miles Herkenham
- Juan Hermoso
- Alejandra Hernández-Agreda
- Felix Hernandez
- Maria Hernandez-Valladares
- Daniel Herranz
- Hector Herranz
- Carolina Herrera
- Carl Herrmann
- Mathias Herrmann
- Cyril Herry
- Jonathan Herskovitz
- Uri Hertz
- Jochen Hess
- Joanna Hester
- Mark Hester
- Ewald Hettema
- Claudio Hetz
- Andreas Heuer
- Volker Heussler
- John Heymach
- Holger Heyn
- Andrew Heyward
- Frankie Heyward
- Clayton Hickey
- Andrés Hidalgo
- Ville Hietakangas
- Tetsuya Higashiyama
- Matt Higgins
- Michael Higgins
- Paul Higgins
- Sarah Highlander
- Allan Hildesheim
- Ryan Hill
- Elisa Hill-Yardin
- Frank Hillary
- Sebastian Hiller
- Angie Hilliker
- Bradley Hillman
- Steve Hillyard
- Julian Hillyer
- Jeroen Hiltermann
- Brett Hilton
- Kristiina Himanen
- Benjamin Himes
- William Hines
- Veronica Hinman
- Winfried Hinrichs
- Hiroyuki Hioki
- Kiyoshi Hirahara
- Jibril Hirbo
- Matthew Hirschey
- Karen Hirschi
- Katherine Hisert
- Matthew Hitchings
- Carl Hjelmen
- Joanne Hobbs
- Luke Hoeppner
- Carsten Hoffmann
- Federico Hoffmann
- Susanne Hoffmann
- Ralf Hofmann
- Zuzana Hofmanová
- Ctirad Hofr
- Michael Hofreiter
- Ursula Hofstötter
- Michael Holbrook
- Arun Holden
- Séamus Holden
- Linda Holland
- Frédéric Hollande
- Marcel Hollenstein
- Bonnie Holmes
- Nicholas Holowka
- Daniel Holstein
- Jeffrey Holt
- Daniela Holtgräwe
- Harry Holthofer
- Andreas Holzinger
- Bernhard Hommel
- Jared Homola
- Yoshiko Honda
- Sang Hong
- Seok Hoon Hong
- Young Bin Hong
- Carina Hoorn
- Adam Hoppe
- Marten Hornsveld
- Mia Horowitz
- Eric Horstick
- Bruce Horwitz
- Akitsu Hotta
- Jin Hou
- Peng Hou
- Jon Houseley
- Dominique Housset
- Henrietta Howells
- Emily Howerton
- Wolfgang Hoyer
- Chih-Hao Hsieh
- Po-Jang Hsieh
- Ching-Lung Hsu
- Shih‐Hsien Hsu
- Bing Hu
- Chang-Deng Hu
- David Hu
- Gangqing Hu
- Guo-Fu Hu
- Kaifeng Hu
- Ping Hu
- Sarah Hu
- Shi Hu
- Xiangming Hu
- Zheng Hu
- Cheng-Yang Huang
- Danny Huang
- Honghui Huang
- Hui Huang
- Kai Huang
- Kuan-Ying Huang
- Paul Huang
- Ruby Huang
- Shanjin Huang
- Shu-Wei Huang
- Shuo Huang
- Y. Mindy Huang
- Yifei Huang
- Yu Huang
- Yun Nancy Huang
- Zirui Huang
- Ziwei Huang
- Jochen Hub
- Thomas Huber
- Clemens Hug
- Konrad Hughen
- Kelly Hughes
- Michael Warren Hughes
- Nigel Hughes
- Randall Hughes
- Leon Hugo
- Lijian Hui
- Mark Huising
- Patrick Humbert
- Emily Humble
- Alistair Hume
- Susanne Hummel
- Louise Humphrey
- Neil Hunt
- Tony Hunter
- Johannes Huppa
- Soojung Claire Hur
- James Hurley
- Jenniver Hurley
- Jane Hurst
- Mushtaq Hussain
- Michael Hust
- Dietmar Hutmacher
- Thomas Hutson
- Carl Hutter
- Mikko Huttunen
- Jennifer Huynh
- Vincent Hyenne
- Chaz Hyseni
- Pirro Hysi
- Ingram Iaccarino
- Alina Iakovleva
- Iskander Ibrahim
- Sherif Abdelaziz Ibrahim
- Mami Iima
- Omkar Ijare
- Dora Il'yasova
- Ray Iles
- Björn Illing
- Natalia Ilyushina
- Hiroo Imai
- Takuya Imai
- Yuuki Imai
- Yoshikazu Imanishi
- Anne Imberty
- Stefano Indraccolo
- Giorgio Innocenti
- Asuka Inoue
- Stefano Ioannucci
- Thoman Ioerger
- Francesco Iorio
- Grzegorz Ira
- Gregorio Iraola
- M. Luisa Iruela-Arispe
- Aaron Irving
- James Irving
- Ehud Isacoff
- Ole Isacson
- Maria Isaguliants
- Brant Isakson
- Sebastian Isbaner
- Joerg Isensee
- Alumit Ishai
- Kazuyoshi Ishigaki
- Md. Shahidul Islam
- Nina Isoherranen
- Raffaella Isola
- Lori Isom
- Philippe Isope
- Rivkah Isseroff
- Kaoru Ito
- Mayumi Ito
- Yutaka Ito
- Giuliano Iurilli
- Larissa Ivanova
- Andrew Iwaniuk
- Smita Iyer
- Tina Izard
- Juan Carlos Izpisua Belmonte
- Alexander Ja Deutsch
- Aaron Jackson
- Andrew Jackson
- Brandon Jackson
- Laura Jackson
- Walker Jackson
- Ralf Jacob
- Jon Jacobs
- Marc Jacobs
- Suzanne R. Jacobs
- Magnus Jacobsen
- Charlotte Jacquemot
- Hans Jacquemyn
- Marie-Agnès Jacques
- Alain Jacquier
- Ramesh Jadi
- Pooja Jadiya
- Amirhossein Jafarian
- Seyed Mehdi Jafarnejad
- Andrew Jaffe
- David Jaffe
- Samie Jaffrey
- Jonathan Jagodnik
- Neda Jahanshad
- Akshay Jain
- Pankaj Jaiswal
- Arjen Jakobi
- Lars Jakobsson
- Matti Jalasvuori
- Oscar Jalnefjord
- Andrew James
- Elizabeth James
- Swadhin Chandra Jana
- Young Jang
- Harald Janovjak
- Ruud Jansen
- Andreas Janshoff
- Li-En Jao
- Phillip Jardine
- Chris Jaroniec
- R. Jarvis
- Rahul Jasrotia
- Sebastien Jauliac
- Jean-Michel Jault
- Joaquim Jaumot
- Marie-Claude Jaurand
- Jonathan Javitch
- Ali Jawaid
- Anass Jawhari
- Murukarthick Jayakodi
- Kaushik Jayaram
- Ali Jazayeri
- Nicholas Jeffery
- Nico Jehmlich
- Gaspar Jekely
- Albert Jeltsch
- Charlie Jennison
- Brian Colwell Jensen
- Just Jensen
- Lasse Jensen
- Mogens Jensen
- Choongwon Jeong
- Robert Jernigan
- Michael Jewett
- Puni Jeyasingh
- Sudhakar Jha
- Suman Jha
- Yachana Jha
- Daoyun Ji
- Guang Ji
- Jiansong Ji
- Lei Ji
- Minbiao Ji
- Honglin Jia
- Zhengping Jia
- Hong-Chen Jiang
- Hua Jiang
- Lei Jiang
- Ning Jiang
- Shuai Jiang
- Wen Jiang
- Xiaosong Jiang
- Yu Jiang
- Ziwen Jiang
- Shuliang Jiao
- Francis Jiggins
- Felix Jimenez-Jimenez
- Suoqin Jin
- Yong-Su Jin
- Robert Jinkerson
- Sarawut Jitrapakdee
- Zerina Johanson
- Erik Johansson
- Jan Johansson
- Sabu John
- Kevin Johnson
- Rachelle Johnson
- Alice Johnston
- Brian Johnston
- Elizabeth Johnstone
- Seetharama Jois
- Mohit Kumar Jolly
- Elizabeth Jones
- Jennifer Jones
- Justin Jones
- Katrina Jones
- Rose Jones
- Teri Jones
- Benedicte Jordan
- Daniel Jordan
- Katherine Jordan
- Peter Jordan
- Maëlle Jospin
- Deirdre Joy
- Sebastian Joyce
- Gabor Juhasz
- Jean-Philippe Julien
- Woo Jun Sul
- Howard Junca
- Mi-Yeon Jung
- Tae Sung Jung
- Ivan Junier
- Loïc Kéver
- Daniel Kümmel
- Balint Kacsoh
- Martin Kaczocha
- Neil Kad
- Amal Kaddoumi
- Takashi Kadowaki
- Erik Kaestner
- Bjorn Kafsack
- Igor Kagan
- Ryoichiro Kageyama
- Luciane Kagohara
- Yang Kai-Chien
- Kai Kaila
- Taku Kaitsuka
- Vladimir Kalinichenko
- Nir Kalisman
- Tuula Kallunki
- Ioannis Kalomenidis
- Rainer Kalscheuer
- Shivani Kamdar
- Ken-Ichiro Kamei
- Laura Kamenetzky
- Juan Kamienkowski
- Ryoma Kamikawa
- Bozena Kaminska
- Clemens Kaminski
- Rafal Kaminski
- Hyunseok Kang
- Sumin Kang
- Tae Hyun Kang
- Un Jung Kang
- Ram Kannan
- Thirumala-Devi Kanneganti
- Ronan Kapetanovic
- Karen Kapheim
- Gisela Kaplan
- T. Joseph Kappock
- Aphrodite Kapurniotu
- Erkan Karakas
- Canan Karakoç
- Sherif Karama
- John Karas
- Eva Karasmanis
- Jason Karch
- Narain Karedla
- Michael Kareta
- Petr Karlovsky
- Georgia Karpathiou
- Sandor Kasas
- Fatah Kashanchi
- Matthias Kashube
- Maria Kasper
- Ksenia Kastanenka
- Zaneta Kaszta
- Vladimir Katanaev
- Hideaki Kato
- U. Benjamin Kaupp
- Kaveh Kavousi
- Masayoshi Kawaguchi
- Shigeyuki Kawai
- Taro Kawai
- Koichi Kawakami
- Emily Kawaler
- Izuru Kawamura
- Hisashi Kawasaki
- Kazuhiko Kawasaki
- Takumi Kawasaki
- Toshimitsu Kawate
- Brian Kay
- Hajime Kayanne
- Józef Kazmierczak
- Yonggang Ke
- Michael Kearney
- Robert Keenan
- Vladimir Kefalov
- Shella Keilholz
- Erin Kelleher
- Christopher Kello
- John Kelly
- Libusha Kelly
- Roy Kelner
- Max Kelz
- Ildiko Kemenes
- Sharon Kendall
- Marc Kenis
- Ann Kennedy
- Kenry Kenry
- Alex Kentsis
- David Kerns
- Bethany Kerr
- Emma Kerr
- Nagaraj Kerur
- Ali Keshavarzian
- Nicoletta Kessaris
- Tijs Ketelaar
- Mireille Khacho
- Afshin Khadangi
- Annette Khaled
- Walid Khaled
- Bahia Khalfaoui-Hassani
- Mehdi Khamassi
- Bilon Khambu
- Kabir Khan
- Nafees Khan
- Anupama Khare
- Anthony Khawaja
- Roustem Khazipov
- Mazen Kheirbek
- Elena Khlestkina
- Saadi Khochbin
- Dion Khodagholy
- Kay-Hooi Khoo
- Chiea Chuen Khor
- Habibeh Khoshbouei
- Pegah Khosravi
- Konstantin Khrapko
- Namrata Khurana
- Jeff Kidd
- Bruno Kieffer
- Daniel Kierzkowski
- Stefan Kiesgen
- Anna Kietrys
- Tim Kietzmann
- Takanori Kigawa
- Mogens Kilian
- Halil Kilicoglu
- Betty Kim
- Boa Kim
- Bong-Soo Kim
- Byoung Chan Kim
- Daehwan Kim
- Dongsup Kim
- Hajin Kim
- Jae Bum Kim
- James Kim
- Jonggul Kim
- Jongkee Kim
- Junhwan Kim
- Juno Kim
- Min-Ho Kim
- Namshin Kim
- Seungill Kim
- Taeyoon Kim
- Yong-Mi Kim
- Yong-Sam Kim
- Michael King
- Paul Kingsley
- Stuart Kininmonth
- Tyler Kirby
- Elsa Kirchner
- Natalia Kirienko
- Clare Kirkpatrick
- Alfredo Kirkwood
- Joanna Kirman
- Lorrie Kirshenbaum
- Anish Kirtane
- David Kissick
- Joseph Kissil
- Harold Kistler
- Daiju Kitagawa
- Toshimori Kitami
- Takashi Kitamura
- Eugene Kiyatkin
- Hiroaki Kiyokawa
- Kazuma Kiyotani
- Shinae Kizaka-Kondoh
- Rainer Klages
- Susana Brom Klanner
- Christian Klein
- Elise Klein
- Ramon Klein Geltink
- Miriam Klein-Flugge
- Tatjana Kleine
- Sabine Kleinsteuber
- Rachel Klevit
- Jitka Klimešová
- William Klimstra
- David Kline
- Adam Klosin
- Anke Klueter
- Christian Klug
- Judith Klumperman
- Rob Knell
- Vincent Knight-Schrijver
- Allison Knoll
- Eileen Knorr
- Gavin Knott
- Erik Knudsen
- Jaewon Ko
- Eva Kočišová
- Ken Kobayashi
- Stephan Koblmüller
- Martina Kocan
- Christian Koch
- Hans‐Georg Koch
- Kamila Kochan
- John Kocik
- Ullasa Kodandaramaiah
- Alwin Koehler
- Geoff Koehler
- Darius Koester
- Eric Kofoed
- Jun-Ichiro Koga
- Kyunghee Koh
- Michael Kohl
- Annegret Kohler
- Ai Koizumi
- Michal Kolář
- Evangelos Kolettas
- Venkatesh Kolluru
- Harald Kolmar
- Petros Kolovos
- Natalia Komarova
- Toshihisa Komori
- Kunio Kondoh
- Olga Kondrashova
- Sam-Geun Kong
- Xiangjun Kong
- Hiroyuki Konishi
- Marina Konopleva
- Maria Kontaridis
- Susanne Kooistra
- Gregory Korbutt
- Christoph Korn
- Kseniya Korobchevskaya
- Sigrun Korsching
- N.M. Korthagen
- David Koslicki
- Ioly Kotta-Loizou
- Tilman Kottke
- Leah Kottyan
- Konstantinos Koudounas
- Fani Koukouli
- Marios Koutsakos
- Christos Koutsarnakis
- Dima Kozakov
- Pawel Kozielewicz
- Julia Kozlitina
- Marcus Krüger
- Michael Krützen
- Michael Kracht
- Alwin Kraemer
- Fred Kramer
- Florian Krammer
- Sven Kranz
- Christopher Krapu
- Katon Kras
- W. Lee Kraus
- Kirsten Krause
- Lauren Krausfeldt
- Daniel Kraut
- Jakub Kreisinger
- Simon Kretschmer
- Siddarth Krishnan
- Oleg Krishtal
- Abhichart Krissanaprasit
- David Krist
- Andrei Krivtsov
- Nils Kroemer
- Christopher Kroenke
- John Krolewski
- Emil Kromann
- Johannes Kromdijk
- Gabriel Krouk
- Stefan Kubicek
- Terrance Kubiseski
- Takanori Kubo
- Yoshihiro Kubo
- Naoto Kubota
- Shafi Kuchay
- Vijay Kuchroo
- Tetsuhiro Kudoh
- Misha Kudryashev
- Donald Kufe
- Martin Kuhlwilm
- Angela Kuhn
- Michael Kuhn
- Richard Kuhn
- Jonas Kuiper
- Athan Kuliopulos
- Urmila Kulkarni-Kale
- Ashutosh Kumar
- Janesh Kumar
- Ritesh Kumar
- Saroj Kumar
- Sushil Kumar
- Vibhor Kumar
- Vivek Kumar
- Yoshinori Kumazawa
- Kazuhiko Kume
- Che-Pei Kung
- Gary Kunkel
- Krushnamegh Kunte
- Hiroshi Kunugi
- Morito Kurata
- Jonathan Kurie
- Daisuke Kurihara
- Alexander Kurilshikov
- Nicholas Kurniawan
- Deborrah Kurrasch
- Jochen Kursawe
- Courtney Kurtz
- Takehiro Kusakabe
- Steven Kushner
- George Kustatscher
- Zoltan Kutalik
- Nobuyuki Kutsukake
- Kazuhiko Kuwahara
- N. Muge Kuyumcu-Martinez
- Andrea Kwakowsky
- Kenneth Kwan
- Sze Ting Kwan
- Hang Fai Kwok
- Peter Kwong
- Natasha Kyprianou
- Vasileios Kyttaris
- Erika López Arribillaga
- Hernán López-Schier
- Jan Lünemann
- Martina Jones
- Damien Laage
- Alec Lackmann
- Benoit Lacombe
- Alexey Ladokhin
- Michael Ladomery
- Anne Lagendijk
- Jinsheng Lai
- Ren Lai
- Alessandro Laio
- Patrick Lajoie
- Peter Lakatos
- Seema Lakdawala
- Spencer Lake
- Jeremy Lakey
- Aparna Lakkaraju
- Girdhari Lal
- Peeyush Lala
- Ryan Lalumiere
- Hon-Ming Lam
- John Lamattina
- Sander Lamballais
- Jean-Philippe Lambert
- Nevin Lambert
- Olivier Lambert
- David Lambright
- Meindert Lamers
- Wouter Lamers
- Raphael Lamprecht
- Dimitrios Lamprou
- Fei Lan
- Hui Yao Lan
- Henrik Land
- Ulf Landegren
- Erin Landguth
- Marene Landström
- David Lane
- Paul Lane
- Stephen Lane
- Ursula Lang
- Philipp Lange
- Ellen Langer
- Nicolas Langer
- Lucia Languino
- Connor Lantz
- Andrés Lanzós
- Francesco Lanza
- Michael Lanzer
- Chiara Lanzuolo
- Caroline Laplante
- Dennis Lapuente
- Carolyn Larabell
- Matthew Larcombe
- Maximilian Larena
- Bret Larget
- Irina Larina
- Christopher Larock
- Yolanda Larriba
- Melinda Larsen
- Erik Larsson
- H. Peter Larsson
- Hans Larsson
- Janine Lasalle
- Andrew Lassar
- Peter Latham
- Richard Lathe
- Hélène Launay
- Mélanie Laurin
- Paul Lavender
- Gregory Lavieu
- Martin Lavin
- Antonina Lavrentieva
- Inna Lavrik
- Igor Lavrov
- Matthew Law
- A. Michelle Lawing
- Devon Lawson
- Olga Lazareva
- Beth Lazazzera
- Duc Le
- Lionel Le Bourhis
- Yann Le Guen
- Corentin Le Magueresse
- Claire Le Pichon
- Karine Le Roch
- David Lea-Smith
- Katie Leach
- Richard Leapman
- Francesca Leasi
- Guillaume Lebon
- Maël Lebreton
- Clotilde Lecrux
- Emilia Lecuona
- Albert Lee
- Andrea Lee
- Chia-Hsueh Lee
- Dae-Hee Lee
- Esther Lee
- Hunjoong Lee
- Inah Lee
- Jae-Seong Lee
- Jooyoung Lee
- Junmin Lee
- Katie Lee
- Kyongbum Lee
- L. James Lee
- Min Gyu Lee
- Se-Jin Lee
- Seung Hwan Lee
- Soyoung Lee
- Taek Soon Lee
- Rob Leech
- Susan Lees-Miller
- Hakon Lefler
- Roger Lefort
- Mark Lefsrud
- Megan Leftwich
- Frederic Legendre
- Gaelle Legube
- Michael Lehmann
- Tovi Lehmann
- Ben Lehner
- Jessica Lehoczky
- Janne Lehtiö
- Lei Lei
- Christian Leibold
- Josef Leibold
- Lars Leichert
- Matthias Leisegang
- Dario Leister
- Gabriela Leite
- Vitor Leite
- Maria Leite-De-Moraes
- Alexander Leitner
- Stanley Lemon
- Metka Lenassi
- Audrey Lenhart
- Tobias Lenz
- Sigurd Lenzen
- Thomas Leonard
- Roberta Leonardi
- John Leong
- Stefano Leoni
- Anna Leopold
- David Leopold
- Bernd Lepenies
- Konstantin Lepikhov
- Jason Lerch
- Ludmila Leroy
- Paul Lesbats
- Edward Lesnefsky
- Michael Lesser
- Adelaine Leung
- Jeanette Leusen
- Erez Levanon
- Romain Levayer
- Yevgen Levdansky
- Valeria Levi
- Herbert Levine
- Joshua Levitz
- Anna-Liisa Levonen
- Doron Levy
- Eric Lewallen
- Józef Lewandowski
- Shawn Lewenza
- Monika Lewinska
- Patrick Lewis
- Penelope Lewis
- Richard Lewis
- Zachary Lewis
- Jarrod Lewis-Peacock
- Mathew Lewsey
- Klaus Ley
- Brian Leyland-Jones
- Anan Li
- Chang Li
- Christine Li
- Deqiang Li
- Dianfan Li
- Fei Li
- Howard Li
- Jian Jian Li
- Jianchao Li
- Jiliang Li
- Jingyi Jessica Li
- Ling Li
- Liying Li
- Mei Li
- Meng Li
- Mulin Jun Li
- Ning Li
- Peng Li
- Qian Li
- Shanshan Li
- Shaopeng Li
- Shitao Li
- Tuo Li
- Wei Li
- Xiao-Jiang Li
- Xiaoling Li
- Yanchang Li
- Yong Li
- Yonghe Li
- Yuhui Li
- Yun Li
- Yusheng Li
- Zhen Li
- Zhibin Li
- Zongli Li
- Ivan Liachko
- Cui Liang
- Jingjing Liang
- Winnie Liang
- Yanyu Liang
- Yuting Liang
- James Liao
- Jinbao Liao
- Joseph Liao
- Yung-Feng Liao
- Romain Libbrecht
- Pablo Librado
- Diana Libuda
- Benjamin Lichman
- Jonathan Licht
- Wolfgang Liedtke
- Marc Liesa
- Thor-Seng Liew
- Woei Chang Liew
- Symen Ligthart
- Roland Lill
- Laura Lilly
- Chunghun Lim
- Sai-Kiang Lim
- Theam Soon Lim
- Santiago Lima
- Ryan Limbocker
- Chentao Lin
- Chia-Hua Lin
- Haotian Lin
- Hsiang-Yuan Lin
- Hung-Yu Lin
- Jiandie Lin
- John Lin
- Meng Lin
- Shih-Shun Lin
- Xiaolong Lin
- Xin Lin
- Yingxi Lin
- Yu-Chun Lin
- Laetitia Linares
- Jamie Lincoff
- Matthew Lincoln
- Peter Lindblad
- Maurine Linder
- Stefan Linder
- Anke Lindner
- Charlotte Lindqvist
- Eva Lindström
- Emily Lines
- Jiqiang Ling
- Ole Lingjærde
- Peter Linsley
- Guy Lippens
- Colin Lipper
- Zachary Lippman
- Gerald Lipshutz
- Matteo Lisi
- Shawn Little
- Mathew Littlejohn
- Yael Litvak
- Mike Litzow
- Bao Liu
- Bin Liu
- Bo Liu
- Chen Liu
- Chenli Liu
- Dongyan Liu
- Feng Liu
- Haibo Liu
- Hao Liu
- Haoxuan Liu
- Jia Liu
- Jianfeng Liu
- Jinxin Liu
- Juewen Liu
- Jun Liu
- Kangze Liu
- Qian Liu
- Qin Liu
- Quan-Xing Liu
- Renjing Liu
- Ruisheng Liu
- Sheng Liu
- Shiping Liu
- Siqi Liu
- Tao Liu
- Tong Liu
- Xiao Liu
- Xin Liu
- Xio Liu
- Ya-Wen Liu
- Yan Liu
- Yang Liu
- Zhiwei Liu
- Zhiyong Liu
- Andy Liwang
- Timur Liwinski
- Marcela Lizano
- Anne Ljungars
- Catherine Llanes
- Ana Lleo
- Manuel Llinás
- Stephanie Lo
- Jason Locasale
- Daniel Lodge
- Kristopher Lofgren
- Ryan Logan
- Jacelyn Loh
- Po-Ru Loh
- Timothy Lohman
- Ingrid Lohmann
- Josef Loidl
- Martijn Lolkema
- Giovanna Lombardi
- Fanxin Long
- Hannah Long
- John Long
- Allison Lopatkin
- Jose Maria Lopez Ayala
- Diana Lopez-Barosso
- Manuel Lopez-Cabrera
- Purificacion Lopez-Garcia
- Dmitry Los
- Shaun Lott
- Jizhong Lou
- Yonggen Lou
- Swarnali Louha
- Antoine Louveau
- Julien Louys
- Michael Love
- Connie Lovejoy
- Jonathan Lovell
- David Lovinger
- Marta Lovino
- Aloysius Low
- Noel Lowndes
- Jia-Hong Lu
- Kun Ping Lu
- Mingyang Lu
- Yingchang Lu
- Zheng Lu
- Zhi Lu
- Wolfgang Lubitz
- Elizabeth Lucas
- Kelsey Lucas
- Enrico Luchinat
- Alessandro Luciani
- Fabio Luciani
- Vivian Lui
- Dieter Lukas
- Marcin Lukaszewicz
- Maria Lukina
- Victoria Lund
- Christian Lunetta
- Vladimir Lunin
- Oleg Lunov
- Chongyuan Luo
- Hong Luo
- Jian-Hua Luo
- Jie Luo
- Kaijun Luo
- Ming Luo
- Weibo Luo
- Yun Luo
- Pradeep Luther
- Corinne Lutomski
- Svetlana Lutsenko
- Peter Lwigale
- Iseult Lynch
- Kristen Lynch
- Karen Lyons
- Michael Lyons
- Artem Lysenko
- William Lytton
- Dmitry Lyumkis
- Ari Pekka Mähönen
- Miia Mäkelä
- Marlo Möller
- Christian Möllmann
- Susann Müller
- Annette Müller-Taubenberger
- Christian Münch
- Boxue Ma
- Eric Ma
- Jian Ma
- Jianzhu Ma
- Qiufu Ma
- Shuyi Ma
- Tao Ma
- Xiaojing Ma
- Yu-Qiang Ma
- Marzena Maćkowiak
- Mohammad Ma'aitah
- Yuichi Machida
- Maro Machizawa
- David Machugh
- Sara Macias
- Charles Mackay
- Joel Mackay
- Cameron Mackereth
- Annette Macleod
- Lesley Macneil
- Sonya Macparland
- Charlotte Madore
- Katsumi Maenaka
- Steven Maere
- Ignacio Maeso
- Jules Magda
- Alessandro Magli
- Giovanni Maglia
- Enrico Magnani
- Jenny Magnes
- John Maher
- Ahmed Mahfouz
- Magdy Mahfouz
- Moe Mahjoub
- Ana Teresa Maia
- Filipe Maia
- Helder Maiato
- Isabella Maiellaro
- Jessica Maiers
- Bernardo Mainou
- Richard Mains
- Sourav Maity
- Michelle Maiworm
- Barbara Majello
- Sebastian Major
- Sharmistha Majumdar
- Katarzyna Majzner
- Tak Mak
- Wing Cheung Mak
- Takashi Makino
- Tomoko Makishima
- Carolina Makowski
- Hannah Malcolm
- Maria Maldonado
- Pedro Maldonado
- Hélène Malet
- Assumpció Malgosa
- Erich Malkemper
- Krishna Mallela
- James Mallet
- Craig Malloy
- Alexander Malogolovkin
- Stefano Mammola
- Si Ming Man
- Balachandran Manavalan
- Nicholas Mancuso
- Susanne Mandrup
- Giovanni Manfredi
- Serghei Mangul
- Angelika Manhart
- Sridhar Mani
- Ani Manichaikul
- Richard Mann
- Hanna Mannell
- Jaan Mannik
- Michael Manson
- Nahal Mansouri
- Ivan Manzini
- Angelo Mao
- Fangyuan Mao
- Kangshan Mao
- Youdong Mao
- Frederic Maps
- Adam Mar
- Eric Maréchal
- Antonio Maraver
- Claire Marceaux
- Vanessa Marcelino
- D. Blaine Marchant
- Asaf Marco
- Thomas Maresca
- David Margolis
- Abelardo Margolles
- Daniel Margulies
- David Margulies
- Valeria Marigo
- Riccardo Marioni
- Encarnita Mariotti-Ferrandiz
- David Mark Welch
- John Markley
- Giorgos Markou
- Sylvie Marleau
- Ines Marques
- Wesley Marques
- Nicholas Marra
- Gary Marsat
- Deborah Marsh
- Lee Marshall
- Alexander Marson
- Alberto Martín-Serra
- Esperanza Martínez-Romero
- Megan Martik
- Andrew Martin
- Brent Martin
- Carlos Martin
- Craig Martin
- David Martin
- Frank Martin
- Stephen Martin
- T. John Martin
- Ana Martin-Hernandez
- Jose Martinez-Navio
- Alessandra Martini
- Anna Martner
- Venkat Maruthamuthu
- Asier Marzo
- Stefania Masci
- Cecilia Mascolo
- Robin Maser
- Rosalinde Masereeuw
- Ameya Mashruwala
- David Masopust
- Jon Massey
- Dana Mastrovito
- Naoki Masuda
- Ruturaj Masvekar
- Irina Matei
- Joaquin Mateo
- Abed Mathagu
- Marion Mathelié-Guinlet
- Ulrike Mathesius
- Debra Mathews
- Michael Mathews
- Paul Mathews
- Sarah Mathews
- Iain Mathieson
- Anukriti Mathur
- Ramkumar Mathur
- Vartika Mathur
- Takashi Matozaki
- Rebecca Matsas
- Kunihiro Matsumoto
- Shoji Matsumura
- Toshiya Matsushima
- Patrick Matthias
- Jelle Matthijnssens
- Steffi Matthyssen
- Julie Maupin-Furlow
- Francesco Maura
- Andrew Maurer
- Claudia Mauri
- Madelon Maurice
- Harold May
- David Mayerich
- William Mayhan
- Dominique Mazier
- Yuval Mazor
- Raja Mazumder
- Pawel Mazur
- Linas Mazutis
- Roberta Mazzieri
- Pietro Mazzoni
- Alison Mcafee
- James Patterson Mcallister
- Tim Mcallister
- Jordan Mccall
- Ryan Mcclure
- Bruce Mccollister
- Alistair Mccormick
- Chris Mccrum
- David Mcculley
- Megan Mcdonald
- Robert Mcdonald
- Jennifer Mcdonough
- Noel Mcelvaney
- James Mcfarland
- Matthew Mcginley
- Jenny Mcguire
- Leeanne Mcgurk
- Campbell Mcinnes
- Aaron Mckenna
- James Mckenna
- Gian-Luca Mclelland
- Elizabeth Mcmillan
- M. Windy Mcnerney
- Nathan Mctigue
- Gareth Mcvicker
- Maria Medalla
- Diego Medina
- Irene Meester
- Christina Megli
- Shahid Mehmood
- Samina Mehnaz
- Devang Mehta
- Matthias Meier
- Francesco Meinardi
- Silke Meiners
- Joyce Meiring
- Giuseppe Melacini
- Christian Melander
- Carmen Melendez-Vasquez
- Emmanuel Mellet
- Stephano Mello
- Jack Mellor
- Adam Melvin
- Raoul-Martin Memmesheimer
- Fangang Meng
- Jia Meng
- Qinbing Meng
- Weihua Meng
- Benedetta Mennucci
- Mitra Menon
- Alexander Mentzer
- Karen Menuz
- Jean-Louis Mergny
- Felipe Merino
- Christine Merlin
- Rob Mesman
- Enrique Mesri
- Ilhem Messaoudi
- Meredith Metzger
- Kyle Meyer
- Marc Meyers
- Filip Meysman
- Qing-Sheng Mi
- Chris Miall
- Ji Miao
- Yansong Miao
- Silvestro Micera
- Magdalene Michael
- Martin Michaelis
- George Michalopoulos
- J.P. Michaud
- Tatsuo Michiue
- Gracjan Michlewski
- Matthew Mickens
- Stuart Middlemiss
- Michihiro Mieda
- Ainhoa Mielgo
- Adriana Migliorini
- Maria Mihaylova
- Sanja Mikulovic
- Marco Milan
- Dragan Milenkovic
- Dusanka Milenkovic
- Donald Miles
- Jeremy Miller
- Joshua Miller
- Karl Miller
- Steve Miller
- Gordon Mills
- Justin Milner
- Rinki Minakshi
- Akira Mine
- Geltrude Mingrone
- John Minna
- Ony Minoarivelo
- Marcelo Mira
- Masanori Mishima
- Alexander Mishin
- Yuji Mishina
- Dan Mishmar
- Monalisa Mishra
- Padmaja Mishra
- Sabyashachi Mishra
- Bratislav Misic
- Ralph Mistlberger
- Pawel Misztal
- John Mitani
- Angela Mitchell
- Christina Mitchell
- D. Rex Mitchell
- Diana Mitchell
- Antonina Mitrofanova
- Shriyaa Mittal
- John Mittler
- Kenji Miura
- Shinji Miura
- Yuko Miyagoe-Suzuki
- Masahiro Miyake
- Akio Miyamoto
- David Miyamoto
- Makoto Miyara
- Eiichi Mizohata
- Xian Mo
- Thomas Mock
- Helmout Modjtahedi
- Leonhard Moeckl
- Arne Moeller
- Alexander Moffett
- Majid Mohajerani
- Helai Mohammad
- Krithika Mohan
- Wendy Mok
- Dejana Mokranjac
- George-Lucian Moldovan
- Sara Mole
- Isabel Molina
- Miriam Molina Arcas
- Abolfazl Mollalo
- Barbara Molon
- Daniel Monaghan
- Samsuzzoha Mondal
- Santanu Mondal
- Stephen Mondo
- Alexander Mongin
- Carrie Mongle
- Véronique Monnet
- Oscar Monroig
- Antonia Monteiro
- Rui Monteiro
- Christopher Montgomery
- Grant Montgomery
- Stephen Montgomery
- Cedric Montigny
- Cheil Moon
- Yuseok Moon
- Bethany Moore
- Peter Moore
- Pippa Moore
- Stuart Moore
- Torben Moos
- Maximilian Mora
- Thierry Mora
- Hector Mora-Montes
- Joanna Moraczewska
- Janet Moradian-Oldak
- Trevor Moraes
- Pedro Moraes-Vieira
- Aixa Morales
- Rani Moran
- Violaine Moreau
- Kelley Moremen
- Herman Moreno
- Lluis Morey
- Marta Morey
- Lucia Morgado-Palacin
- Michael Morgan
- Diego Morgavi
- Hideki Mori
- Munemasa Mori
- Seiichi Mori
- Benjamin Morillon
- Jumpei Morimoto
- Masahiro Morita
- Yasu Morita
- Helene Morlon
- Anna Moroni
- Nadya Morozova
- Hans Morreau
- Nicholas Morrell
- Edward Morris
- Philip Morris
- Ciaran Morrison
- Nigel Morrison
- Alyssa Morrow
- Michael Morrow
- Danila Moscone
- Gregory Moseley
- Ala Moshiri
- Morongwa Mosothwane
- Emilio Mottillo
- Joana Mourão
- Rafael Moura
- Lionel Mourey
- David Moyes
- Masoud Mozafari
- Jorge Mpodozis Marín
- Barbara Muehlemann
- Elisabetta Mueller
- Eugene Mueller
- Stephen Muench
- Paul Muhindira
- Marianne Muhlebach
- Yukio Mukai
- Arnab Mukherjee
- Shaeri Mukherjee
- Srabani Mukherjee
- Debabrata Mukhopadhyay
- Vijaykumar Muley
- Florian Muller
- Philip Mullineaux
- Barbara Mulloy
- Gerhard Multhaup
- Jeanette Mumford
- Karel Mundnich
- Altar Munis
- Peris Munyaka
- Yasuteru Muragaki
- Minae Mure
- Marta Murgia
- Rodolfo Murillas
- Alex Murphy
- Patrick Murphy
- Regina Murphy
- Robert Murphy
- Sean Murphy
- Timothy Murphy
- William Murphy
- James Murray
- Kevin Murray
- Garib Murshudov
- Mala Murthy
- Dhaarini Murugan
- Gopinath Muruganandam
- Gabriella Musacchia
- Angelika Mustroph
- Felicity Muth
- Suresh Kumar Muthuvel
- Alexander Myburg
- Shreesh Mysore
- Kyungjae Myung
- Bernd Nürnberg
- Raffael Nachbagauer
- Arundhati Nag
- Sahana Nagabhushan Kalburgi
- Andries Nagtegaal
- Adam Naj
- Bijan Najafi
- Yusaku Nakabeppu
- Ichiro Nakagawa
- So Nakagawa
- Takuro Nakagawa
- Kiyohiko Nakamura
- Shuhei Nakamura
- Harikrishna Nakshatri
- Jarlath Nally
- Priyanka Nandakumar
- Anirvan Nandy
- Brent Nannenga
- Honami Naora
- Sahin Naqvi
- Hua Naranmandura
- Akshay Narkar
- Steven Narod
- Carla Nasca
- Mahmoud Nasr
- Melissa Nassif
- Samuel Nastase
- Kent Nastiuk
- Kedar Natarajan
- Rama Natarajan
- Katherine Nathanson
- Richard Naud
- Anton Naumov
- Guillermo Navalon
- Ara Nazarian
- Stephan Nebe
- Andreas Nebenführ
- Leonard Neckers
- Michal Neeman
- Matthew Neiditch
- Matthew Nelsen
- Craig Nelson
- Sacha Nelson
- Jeanne Nerbonne
- Donna Neuberg
- Benjamin Neuman
- Aaron New
- Robert Newberry
- Aaron Newman
- Adam Newton
- Kim Newton
- Phillip Newton
- Dan Nicolau
- Yvain Nicolet
- Qinghua Nie
- Steven Niederer
- Dariusz Niedzwiedzki
- James Nieh
- Barbara Niehoff
- John Nieland
- Ashley Nielsen
- Lars Peter Nielsen
- Peter Nielsen
- Uffe Nielsen
- Jan Niess
- Bart Nieuwenhuis
- Alexey Nikitin
- Eleni Nikolakaki
- Johan Nilvebrant
- Guy Nir
- Noriyuki Nishida
- Makiya Nishikawa
- Riko Nishimura
- Wataru Nishimura
- William Nobis
- William Noble
- Christopher Nobles
- Tatsuya Nobori
- Jean-Paul Noel
- Matthew Nolan
- Wataru Nomura
- Peter Nonacs
- Daan Noordermeer
- Lorena Norambuena
- Erik Norberg
- Trista North
- Fernando Novas
- Dorota Nowak
- Evgeny Nudler
- Muhammad Numan
- Alexandra Nunes
- Ruth Nussinov
- Helen Nuttall
- Janet Nye
- Dale Nyholt
- Alexander Nyström
- Edward O'Brien
- John O'Brien
- Claire O'Callaghan
- Colm O'Huigin
- Cecilia O'Leary
- Jeremy O'Sullivan
- Sinead O'Sullivan
- Ronan O'Toole
- Chris Oakes
- Jenny Oakley
- Carole Ober
- Felix Oberhauser
- Nagahiro Ochiai
- Raúl Ochoa-Hueso
- Matthias Ochs
- Yoshihisa Oda
- Peter Oelschlaeger
- Stefan Offermanns
- Remko Offringa
- Takehiko Ogawa
- Ikuo Ogiwara
- Atsuo Ogura
- Jaehyeon Oh
- Seunghee Oh
- Misato Ohtani
- Shigetoshi Oiki
- Hirokazu Okada
- Ui Okada
- Takaharu Okajima
- Kenji Okamoto
- Hirohiko Okamura
- Yasushi Okamura
- Hideyuki Okano
- Freyja Olafsdottir
- Anthony Olarerin-George
- Monica Oleastro
- Łukasz Olewnik
- Meritxell Oliva
- Vani Oliveira, Jr.
- Le Meur Olivier
- Lars Rønn Olsen
- Shaun Olsen
- Michael Olson
- Kyla Omilusik
- Jose Onata-Garzon
- Brian Ondov
- Ju Lynn Ong
- Daisuke Ono
- Noriaki Ono
- Shoichiro Ono
- Raymond Oomen
- Ryoko Oono
- Chris Oostenbrink
- Michael Opata
- Enrico Opri
- Richard Oram
- Steve Ormerod
- William Orsi
- Caitlin Orsini
- Marion Orsucci
- Joaquín Ortega Castro
- Isamar Ortiz-Rivera
- Jesus Osada
- Hiroyuki Oshiumi
- Quinn Ostrom
- Shuhei Ota
- Martin Ott
- Cees Otto
- Oliver Otto
- Le Ou-Yang
- Malika Oubaha
- Aukje Oudelaar
- Judit Ovadi
- Scott Owen
- Gregory Owens
- Philip Owens
- George Owttrim
- Michiko Oyoshi
- Andres Ozaita
- Aydogan Ozcan
- Engin Ozcivici
- Jesús Pérez-Gil
- Silvia Pérez-Lluch
- Martin Pšenička
- Georg Pabst
- Valentina Pacella
- Maurizio Pacifici
- Pablo Paez
- Jesús Page
- Annie Page-Karjian
- Joy Pai
- Sachin Pai
- Joris Paijmans
- Kostandin Pajcini
- Janelle Pakan
- Bhupinder Pal
- Sri Paladugu
- Murugesan Palaniappan
- Iam Palatnik de Sousa
- Gottfried Palm
- Magnus Palmblad
- Ann Palmenberg
- Amy Palmer
- Colin Palmer
- Gregory Palmer
- Stefano Palminteri
- Peter Palukaitis
- Satu Palva
- Ian Pan
- Min-Hui Pan
- Xiaoyong Pan
- Xiaoyue Pan
- Yufeng Pan
- Zhicheng Pan
- Kaushik Panda
- Santosh Panda
- Veethika Pandey
- Rucha Pandit
- Daxin Pang
- Shanshan Pang
- Stanley Pang
- Yue Pang
- Pierpaolo Pani
- Stanislava Pankratova
- Nick Pannunzio
- Periklis Pantazis
- Kostas Pantopoulos
- Pierre Paoletti
- Konstantinos Papadimitriou
- Thales Papagiannakopoulos
- Argyris Papantonis
- F. Nina Papavasiliou
- Jason Papin
- Narayanan Parameswaran
- Efrosyni Paraskeva
- Juan Pablo Pardo
- Heath Pardoe
- Kristin Parent
- Jeehye Park
- Joon Seok Park
- Kyung-Soon Park
- Seong Hwan Park
- Tae-Yoon Park
- Woong-Yang Park
- Young-Kyoung Park
- Dane Parker
- Hugo Parker
- John Parkinson
- Adriana Parodi-Talice
- Rajesh Parsanathan
- Maddy Parsons
- Raghavendran Partha
- Anthony Partridge
- Annette Paschen
- Ben Pascoe
- Francesco Pasqualini
- Ievgenia Pastushenko
- Ann-Marie Patch
- Dhaval Patel
- Smita Patel
- Sushant Patil
- Etienne Patin
- Julian Paton
- Julian Paton
- Karen Patterson
- Hemant Paudel
- Anirban Paul
- Keryn Paul
- Perrine Paul-Gilloteaux
- Ika Paul-Pont
- Ian Paulsen
- James Paulson
- Robert Paulson
- Kajsa Paulsson
- Elizabeth Pavez Lorie
- Youri Pavlov
- Magdalena Pawełkowicz
- Lindsay Payer
- Luz Paz-Maldonado
- Robert Pazdro
- Fernando Peña Ortega
- Claire Peart
- Barrie Peck
- Jean-Denis Pedelacq
- Ole Pedersen
- Christopher Pedigo
- Anthony Pedley
- Isabela Pedroza-Pacheco
- Duanqing Pei
- Michael Peitz
- Serge Pelet
- Caroline Pellet-Many
- Nieves Peltzer
- Lianwei Peng
- Zhihai Peng
- Giusy Pennetta
- Trevor Penning
- Martin Pera
- Michele Perazzolli
- Paris Perdikaris
- Alberto Pereda
- Ana Marta Pereira
- Bruno Pereira
- Camilo Perez
- Mileidys Perez-Alea
- Miguel Perez-Enciso
- Rolando Perez-Lorenzo
- Mariel Perez-Zabaleta
- David Perlmutter
- Fabiana Perocchi
- George Perry
- Mihaela Pertea
- Matthew Perugini
- Kalliopi Pervolaraki
- Mathias Pessiglione
- Luiz Pessoa
- Jason Peters
- Ulrike Peters
- Daniel Peterson
- Matt Peterson
- Ezequiel Petrillo
- Konstantinos Petritis
- Sari Peura
- Benjamin Pey
- Gerald Pfeffer
- Suzanne Pfeffer
- Brad Pfeiffer
- Otto Phanstiel
- Koenraad Philippaert
- Julie Phillippi
- Nancy Philp
- Anne Piantadosi
- Gary Piazza
- Didier Picard
- Martin Picard
- Mathieu Picardeau
- Pietro Picconi
- Nicole Piche-Nicholas
- Hilda Pickett
- Silvia Piconese
- Birgit Piechulla
- Gary Pielak
- Karen Pierce
- Małgorzata Pierzchalska
- Eric Pietras
- Pim Pijnappel
- Teuta Pilizota
- Shiv Pillai
- Viju Vijayan Pillai
- Nicolas Pilon
- Dan Pina Fuentes
- Fabien Pinaud
- Esther Pinheiro
- Mariana Pinho
- Itziar Pinilla-Macau
- Stefan Pinter
- Paola Piomboni
- Andreas Pircher
- Rosario Piro
- Liise-Anne Pirofski
- Angelo Pirrone
- Antonio Pisani
- Michael Pittman
- Mara Pitulescu
- Javier Pizarro-Cerda
- Andres Pizzorno
- Allen Place
- Anna Planas
- Germán Plata
- Michelina Plateroti
- Michael Plavcan
- Begoña Lavin Plaza
- Gustavo Plaza
- Juergen Plitzko
- Jonathan Ploski
- Fabian Plum
- Ross Poché
- Luka Pocivavsek
- Christine Podrini
- Alessandro Poggi
- Mikail Pogorelyy
- Claudia Pogoreutz
- Alexander Pohle
- Laurent Poirel
- Bogdan Polevoda
- Omero Poli-Neto
- Jonathan Polimeni
- Shaul Pollak
- Tim Pollex
- Simona Polo
- Luca Polonio
- Palmiro Poltronieri
- Patrizia Polverino De Laureto
- Frederic Poly
- Véronique Pons
- Gabriel Popescu
- Brian Popko
- Vencislav Popov
- Marcin Poreba
- Dipak Poria
- Stephen Poropat
- Eduard Porta
- James Porter
- Douglas Portman
- Helmut Pospiech
- Danielle Posthuma
- Catherine Postic
- Emmanuel Pothos
- Marie-Claude Potier
- Matthew Potthoff
- Thomas Poulos
- Yves Poumay
- Celio Pouponnot
- Jennifer Powell
- Jen Poynter
- Amynah Pradhan
- Simant Prakoonwit
- Ashok Prasad
- Kalika Prasad
- Kari Prassack
- Joana Prata
- Erica Pratt
- Clarissa Prazeres Da Costa
- Rytis Prekeris
- Tino Prell
- Marc Prentki
- Cindy Prescott
- Diego Presman
- Avi Priel
- Lawrence Prince
- Daniel Principe
- Sarah Pringle
- Fernanda Priviero
- Sambhawa Priya
- Tassula Proikas-Cezanne
- Andreas Prokesch
- Andreas Prokop
- Ludmila Prokunina-Olsson
- Andrey Przhibelsky
- Nikolaos Psonis
- Thomas Pucadyil
- Holger Puchta
- Boas Pucker
- Raffaelo Pugliese
- Elisabetta Viani Puglisi
- Cristina Puig-Saus
- Erdem Pulcu
- Sara Pulit
- Enkhsaikhan Purevjav
- Vishwajeet Puri
- Rahul Purohit
- Michael Purugganan
- Kirstin Purves
- Andrey Puzachenko
- Anna Pyle
- Mirza Muhammad Fahd Qadir
- Chao-Nan Qian
- Jiang Qian
- Xuyu Qian
- Zhaohui Qian
- Hongmin Qin
- Jun Qin
- Jiangping Qiu
- Lili Qiu
- Xiao-Bo Qiu
- Yin-Long Qiu
- Loredana Quadro
- Tiago Quental
- Ethel Queralt
- Corbin Quick
- Jason Quinn
- Kyle Quinn
- Christian Rödelsperger
- Lisa Röttjers
- Nancy Raab-Traub
- Ton Rabelink
- Prakash Radhakrishnan
- Zoran Radić
- Lilliana Radoshevich
- Grazia Daniela Raffa
- Laura Raffield
- Laura Ragona
- Osama Rahma
- Mohammed Nadjib Rahmoun
- Kunal Rai
- Amanda Raine
- Joe Rainger
- Candace Raio
- Daniela Rajão
- Sudarshan Rajagopal
- Priya Rajarapu
- Satyaki Prasad Rajavasireddy
- Nagarajan Raju
- Daria Rakitina
- Sabyasachi Rakshit
- Peter Ralph
- Markus Ralser
- Prakash Ramachandran
- Saravana Ramasamy
- Pavan Ramdya
- Pranela Rameshwar
- Jose Ramirez
- Daniela Ramos
- Elena Rampanelli
- Tharmarajan Ramprasath
- Carly Randall
- Christopher Ranger
- Kishu Ranjan
- Abhiram Rao
- Sneha Rao
- Venigalla Rao
- Xiancai Rao
- Yu Rao
- Rino Rappuoli
- Aaron Rashotte
- Jevgenij Raskatov
- Torben Rasmussen
- Minoo Rassoulzadegan
- Wioletta Ratajczak-Wrona
- Terje Raudsepp
- Michael Rauh
- Martina Rauner
- Sarah Rauscher
- Catherine Ravel
- Raimond Ravelli
- Enrico Ravera
- Maddaly Ravi
- Thirupathi Ravula
- William Rawlinson
- Saikat Ray
- Sumanta Ray
- Julie Rayes
- Kasie Raymann
- Jennifer Raymond
- Pamela Raymond
- Renee Read
- Arran Reader
- Eamonn Reading
- Clare Rebbeck
- Helena Rebelo-De-Andrade
- Ignacio Rebollo
- Juan Recio
- Sarah Reece
- Helen Rees
- Roberto Refinetti
- Thomas Reh
- Tobias Reiff
- Håkon Reikvam
- Shannon Reilly
- Jüri Reimand
- John Reinfelder
- Xiaojun Ren
- Thibaud Renault
- Carlo Renieri
- Nicolas Reynoird
- Ki-Jong Rhee
- Kunsoo Rhee
- Sung-Keun Rhee
- Carmela Rianna
- António Ribeiro
- Gavin Rice
- Denis Richard
- Peter Richard
- Stephen Richards
- Tom Richards
- David Richardson
- Rodney Richardson
- Sara Richter
- Joan Richtsmeier
- Peleg Rider
- Diana Riebold
- Christiane Riedel
- Bernd Rieger
- Jochem Rieger
- Simone Riehl
- Olaf Riess
- Isidore Rigoutsos
- Jason Rihel
- Lisa Riley
- Jasper Rine
- Harald Ringbauer
- Malene Ringkjøbing Jensen
- Maria Giovanna Riparbelli
- Nils Risgaard-Petersen
- Benjamin Risse
- Olivia Rissland
- Yair Rivenson
- Julio Rivera
- Silvio Rizzoli
- Lawrence Rizzolo
- Trevor Robbins
- Elisha Roberson
- James Roberts
- Lucy Roberts
- Stefan Roberts
- Wade Roberts
- Robert Robey
- Jennifer Rocca
- Pedro Roda-Navarro
- M. Rosario Rodicio
- Marina Rodnina
- Aldo Rodríguez-Menchaca
- Vinícius Barros Rodrigues
- Karl Rodriguez
- María Eugenia Rodríguez
- Juan Rodriguez-Flores
- Heriberto Rodriguez-Martinez
- Sylwia Rodziewicz-Motowidło
- Thomas Roeder
- Ofer Rog
- Jeffrey Rogers
- Tim Rogers
- Connor Rogerson
- John Rohde
- Ignasi Roig
- Laila Roisman
- Sofia Roitman
- Charlotte Rolny
- Richard Roman
- Adriana Romero-Olivares
- Ze'ev Ronai
- Shi Song Rong
- Nicola Rooney
- David Root
- Maria Ros
- German Rosano
- Viviana Rosati
- Evan Rosen
- Evan Rosenberg
- Yaeli Rosenberg
- Kobi Rosenblum
- Peter Rosenthal
- Ephron Rosenzweig
- Antonia Roseweir
- Leah Rosin
- Andrew Ross
- Corrina Ross
- Stephan Rosshart
- Barak Rotblat
- Gideon Rothschild
- Rodney Rothstein
- Giovanni Roti
- Veerle Rottiers
- Brice Rotureau
- Marion Rouault
- Tracey Rouault
- Candice Roufosse
- Isabelle Rouget
- Vassilis Roukos
- Guy Rouleau
- Kyle Roux
- David Rowlands
- Shyamal Roy
- Loic Royer
- Wilfried Rozhon
- Beibei Ru
- Xiong Ruan
- Zheng Ruan
- Sara Ruane
- Angela Ruban
- Peter Rubbens
- Mikail Rubinov
- Rüdiger Rudolf
- David Rueda
- Gennaro Ruggiero
- Kelly Ruggles
- Laura Ruiz-Aceituno
- J.C. Ruiz-Suárez
- Holger Russ
- Alan Russell
- Stephen Russell
- Christopher Russo
- Marco Rust
- Pierre Rustin
- Thomas Rutkowski
- Eva Ruusuvuori
- Joseph Ryan
- Timothy Ryan
- Hyun Mo Ryoo
- Zae Young Ryoo
- Choong-Min Ryu
- Andrey Rzhetsky
- James Sáenz
- Miguel Sánchez-Álvarez
- Francisco Sánchez-Bayo
- Lars Sørensen
- Mette Sørensen
- Juliana Sa
- Hannes Saal
- David Sacks
- Timothy Sackton
- Sepideh Sadaghiani
- Enrique Saez
- Julio Saez-Rodriguez
- Mohammad Reza Safaei
- Sandrine Sagan
- Alvaro Sagasti
- Ronit Sagi-Eisenberg
- Megha Sah
- Mehmet Sahin
- Daisy Sahoo
- Akira Saito
- Takeharu Sakamoto
- Shuzo Sakata
- Lori Sakoda
- Daiju Sakurai
- Hiroaki Sakurai
- Roberta Sala
- Ali Salanti
- Jesus Enrique Salcedo-Sora
- Mohammed Saleem
- Muhammad Saleem
- Gianandrea Salerno
- Mootaz Salman
- Robert Salmond
- Paolo Salomoni
- Davinia Salvachua
- Walter Salzburger
- Juhi Samal
- Ramkumar Sambasivan
- Hassan Samee
- Premila Samuel
- Adriana San-Miguel
- Clara Sanchez
- Carlos Sanchez Sorzano
- M. Virginia Sanchez-Puerta
- Miloslav Sanda
- Theo Sanderson
- Yana Sandlers
- Johanna Sandling
- Ioana Sandu
- Joshua Sanes
- Juan Sanguinetti-Scheck
- Mario Sansone
- Andrea Sant
- Stefano Santabarbara
- Naiara Santana-Codina
- Sabato Santaniello
- Tasha Santiago-Rodriguez
- Luca Santini
- Mafalda Santos
- Gangadhara Sareddy
- Dhifaf Sarhan
- Abhishek Sarkar
- Suparna Sarkar-Banerjee
- Masaki Sasai
- Ken Sasaki
- Kotaro Sasaki
- Goro Sashida
- Anuja Sathe
- Akiko Satoh
- Ansuman Satpathy
- Rita Sattler
- Philippa Sauders
- Diane Saunders
- Jaclyn Saunders
- Giuseppe Sautto
- Cathy Savage-Dunn
- Vincent Savolainen
- Michael Savona
- Jimmy Saw
- Kazunobu Sawamoto
- Stephen Sawiak
- Ian Sayers
- Leonid Sazanov
- Matthew Sazinsky
- Pauline Scanlan
- Eliana Scemes
- Erik Schäffer
- Hermann Schätzl
- Ulrich Schüller
- Volker Schünemann
- Christian Schürch
- Antje Schaefer
- David Schaeffer
- Anett Schallmey
- Paul Schanda
- Daniel Schardosim Calovi
- Eitan Schechtman
- Dirk-Jan Scheffers
- Kristina Scheibe
- David Scheinberg
- Jürgen Scheller
- Simon Scheuring
- Andreas Schiermeyer
- Philipp Schiffer
- Oliver Schilling
- Joshua Schimel
- Paul Schimmel
- Daniel Schindler
- David Schlaepfer
- Erik Schleicher
- Ilme Schlichting
- Tamar Schlick
- Christian Schlieker
- Michael Schlossmacher
- Tilman Schlothauer
- Amy Schmid
- Andreas Schmid
- Harald Schmidt
- Marius Schmidt
- Mathias Schmidt
- Rebecca Schmidt
- Anja Schmidt-Christensen
- Urs Schmidt-Ott
- Emmanuelle Schmitt
- Gerold Schmitt-Ulms
- James Schnable
- Rene Schneider
- Jana Schniete
- David Schnyer
- Kilian Schober
- Nathan Schoettler
- Oscar Schofield
- Ethan Scholar
- Dean Scholl
- Gabriele Schonian
- Martina Schröder
- Thomas Schreiner
- Eric Schreiter
- Hannes Schroeder
- Peter Schuck
- Gerhard Schuetz
- John Schuetz
- Ira Schulman
- Laura Schulz
- Rüdiger Schulz
- Stefan Schulz
- Tim Schulz
- Linus Schumacher
- Ben Schumann
- Florian Schur
- Lici Schurig-Briccio
- Erwin Schurr
- Denis Schwartz
- Jacob Schwartz
- Olivier Schwartz
- Erich Schwarz
- Annalisa Scimemi
- John Scott
- Michael Scullin
- Sara Sdelci
- Yakub Sebastian
- Julie Secombe
- Maria Secrier
- Erik Sedlák
- Frank Seebacher
- Guiscard Seebohm
- Markus Seeger
- Valentina Segura
- Garima Sehgal
- Michael Seid
- Tal Seidel Malkinson
- Michael Seidl
- Shiori Sekine
- Albert Selles
- Rachel Seman-Varner
- Amitava Sengupta
- Arjun Sengupta
- Debarka Sengupta
- Kaushik Sengupta
- Kheya Sengupta
- Neelanjana Sengupta
- Jeong-Sun Seo
- Edgardo Sepulveda
- Maria Luisa Sequeira-Lopez
- Ahmet Sertcelik
- Palle Serup
- Somasekar Seshagiri
- Ole-Morten Seternes
- Carmine Settembre
- David Seung
- Claire Sexton
- Emilie Seydoux
- Mathew Seymour
- Erdinc Sezgin
- Eleonora Sforza
- Shabnam Shaabani
- Sergey Shabala
- Sulman Shafeeq
- William Shafer
- Mala Shah
- Shalin Shah
- Yousif Shamoo
- Hongyan Shan
- Shu-Ou Shan
- Yibing Shan
- Peng Shang
- Changwei Shao
- Jian-Zhong Shao
- Mikhail Shapiro
- Rebecca Shapiro
- Robert Shapley
- Abhay Sharma
- Amit Sharma
- Ankush Sharma
- Pushkar Sharma
- Shalini Sharma
- Sudarshana Sharma
- Andrew Sharott
- Paul Sharp
- Prasad Shastri
- Alexander Shaw
- Helen Alexandra Shaw
- Philip Shaw
- Vasu Sheeba
- Medha Shekhar
- Alexander Shekhtman
- Ganesh Shelke
- Tatyana Shelkovnikova
- Scarlet Shell
- Efrat Shema
- Wen Hui Shen
- Xilin Shen
- Xiling Shen
- Yi Shen
- Emily Shepard
- Carmen Sheppard
- Rachel Sherman
- David Sherry
- Guo-Ping Shi
- Huwenbo Shi
- Jianxin Shi
- Kilin Shi
- Qiong Shi
- Shuo Shi
- Tujin Shi
- Shigeru Shibata
- Andy Shih
- Hong-Yan Shih
- Joon Shim
- Sang-Hee Shim
- Fumi Shima
- Jeffrey Shima
- Dmitriy Shin
- Junha Shin
- James Shine
- Elena Shklovskaya
- Maya Shmulevitz
- Osami Shoji
- Sarah Shomstein
- Min Ju Shon
- Dilip Shrestha
- Nick Shrine
- Abhishek Shrivastava
- Wenqing Shui
- Arun Shukla
- Brian Shy
- Jingna Si
- Weena Siangproh
- Andriy Sibirny
- Frank Sicheri
- Bettina Siebers
- Janet Siebert
- Peter Siegel
- Kellee Siegfried
- Kimberly Siegmund
- Evan Siemann
- Rasmus Siersbaek
- Nadejda Sigal
- Sara Sigismund
- Andrew Sih
- Katarzyna Sikora
- David Sillam-Dussès
- Osmar Nascimento Silva
- Ludovico Silvestri
- Daniele Silvestro
- Anton Simeonov
- Linda Simmler
- Steven Simmons
- Rafel Simo
- Marcos Simoes-Costa
- Itamar Simon
- M. Celeste Simon
- Thomas Simon
- Michael Simone-Finstrom
- Jean Simonnet
- Kai Simons
- Matthew Simpson
- David Sinclair
- Charlotte Sinding
- Amar Singh
- Ankita Singh
- Appu Singh
- Ishwar Singh
- Karam Singh
- Nagendra Singh
- Parvesh Singh
- Praveen Singh
- Ramandeep Singh
- Yogesh Singh
- Amit Singhal
- Debasish Sinha
- Sanjay Sinha
- Sanjiv Sinha
- Saurabh Sinha
- Musalula Sinkala
- Herman Sintim
- Konstantinos Siontis
- Supaart Sirikantaramas
- Nicholas Sisco
- Jacobo Sitt
- Sivaraj Sivaramakrishnan
- Harry Siviter
- Łukasz Skalniak
- Elizabeth Skellam
- Laurits Skov
- Christian Skovsted
- Georgios Skretas
- Vasilisa Skvortsova
- Nikolai Slavov
- Stephen Sligar
- Andrii Slonchak
- Susan Slovin
- Igor Slukvin
- Fatima Smagulova
- Ian Small
- Heather Smallwood
- Younes Smani
- Kristina Smiley
- Sandra Smink
- Roelof Smit
- Amy Smith
- Ann Smith
- Edward Smith
- Jeffrey Smith
- Jeramiah Smith
- Keriayn Smith
- Micholas Smith
- Nicola Smith
- Susan Smith
- Sylvia Smith
- Daniel Smith-Paredes
- Sander Smits
- Michael Smotherman
- Joseph Snowden
- Hon-Cheong So
- Ruben Soares
- Alexei Soaresi
- Alexandra Sobeck
- Kee Hoon Sohn
- Nick Sokol
- Shyam Solanki
- Ken Solt
- David Somers
- Luba Sominsky
- Martha Sommer
- Hojun Song
- Wantong Song
- Yujun Song
- Pierre Sonveaux
- Alice Soragni
- Hermona Soreq
- Teiji Sota
- Javier Sotillo
- Mikle South
- Andrew Southam
- Marta Souza
- Sheila Spada
- Anja Spang
- Paul Spearman
- Doug Speed
- Thomas Speltz
- Andrew Spence
- Nicholas Spencer
- Daan Speth
- Robert Spicer
- Diana Spiegelberg
- Ziv Spiegelman
- Eric Spierings
- Hergen Spits
- Cassandra Spracklen
- Thomas Sprague
- Stefania Spranger
- David Spray
- John Spudich
- Pirjo Spuul
- Georgios Spyroulias
- Akshay Sridhar
- Hari Sridhar
- Mandyam Srinivasan
- Anna Stöckl
- Jeanne Stachowiak
- Daniel Stadlbauer
- Rick Stafford
- Christopher Staiger
- Jochen Staiger
- Jason Stajich
- Michael Stallcup
- Stefan Stamm
- Danielle Stanisic
- Nicola Stanley-Wall
- Phillip Stansfeld
- Patric Stanton
- Philipp Starkl
- Justin Stebbing
- Jacob Steenwyk
- Bruce Stein
- Gary Stein
- Lincoln Stein
- Phyllis Stein
- Timo Stein
- Andrea Steinbicker
- Jan SteinküHler
- Stefan Stender
- Monika Stengl
- Alexey Stepanov
- Flora Stephano
- Andrew Stephens
- Jared Sterneckert
- Nicola Stevens
- Angela Stevenson
- Andrew Stewart
- Elizabeth Stewart
- Jason Stewart
- Rodney Stewart
- Sheila Stewart
- Jacob Stewart Ornstein
- Josef Stiegler
- Christian Stock
- Markus Stoffel
- Sanja Stojanovic
- Lovorka Stojic
- Jonathan Stokes
- Emily Stone
- Laura Stone
- Martin Stone
- Kathleen Stoof-Leichsenring
- Mark Stopfer
- Maria Patrizia Stoppelli
- Peter Storz
- Paul Stoy
- Mathew Stracy
- Marie Strader
- Constantine Stratakis
- Timothy Strauman
- Wolfgang Streit
- Sigmar Stricker
- Karin Strijbis
- Astrid Stronen
- Aaron Struck
- Natalie Strynadka
- Anna Sturrock
- Chih-Ying Su
- Xin-Zhuan Su
- Xuantao Su
- Yu-Lin Su
- Elavarasan Subramani
- Ramadevi Subramani Reddy
- Carmen Sucharov
- Kumarasamy Sudhakar
- Makoto Suematsu
- Hans-Dieter Sues
- Fumiaki Sugahara
- Lauren Alpert Sugden
- Yukihiko Sugimoto
- Minetaka Sugiyama
- Rachael Sumner
- Colin Sumrall
- Gui-Quan Sun
- James Sun
- Jianjun Sun
- Junsong Sun
- Ranfeng Sun
- Ruping Sun
- Shisheng Sun
- Thomas Yang Sun
- Yinyan Sun
- Yu Sun
- Subramanian Sundharraman
- Hoon-Ki Sung
- Francesco Sunseri
- Megan Supple
- Kushal Suryamohan
- Cory Suski
- J. Mark Sutton
- Haruo Suzuki
- Masatoshi Suzuki
- Norio Suzuki
- Shinsuke Suzuki
- Maxim Svetlov
- Karel Svoboda
- Vinay Swaminathan
- Fredrik Swartling
- Daan Swarts
- Stephen Swearer
- Trevor Sweeney
- Michael Sweet
- Pawel Swietach
- Lykke Sylow
- Lorraine Symington
- Christopher Synatschke
- Karl Syson
- Anna Szécsényi-Nagy
- Bence Szalai
- Andrzej Szewczak-Harris
- Grazyna Szklarz
- Francisco Taberner
- Arantxa Tabernero
- Mohammad Taghdisi
- Maurizio Taglialatela
- Enzo Tagliazucchi
- Leon Tai
- T.C. Tai
- Yu-Tzu Tai
- Emad Tajkhorshid
- Ayato Takada
- Daisuke Takagi
- Hiroaki Takagi
- Masahide Takahashi
- Keizo Takao
- Takeshi Takarada
- Tetsuo Takehara
- Hiromasa Takemura
- Kenjiro Takemura
- Mizuki Takenaka
- Toru Takimoto
- Frank Takken
- Keiyo Takubo
- Idan Tal
- Yuen Yi Tam
- Arielle Tambini
- Marco Tamietto
- Gintautas Tamulaitis
- Hiroshi Tamura
- Rodrigo Tamura
- Hsih Yin Tan
- Meng How Tan
- Shyh-Han Tan
- Dean Tang
- Hong-Wen Tang
- Kam Tang
- Matthew Tang
- Qi-Qun Tang
- Qinggong Tang
- Pradeep Tanwar
- Rui Tao
- Yi Tao
- Julio Tapia
- Justin Taraska
- Nadya Tarasova
- Eric Tartour
- Jörg Tatzelt
- Manabu Taura
- Nektarios Tavernarakis
- Cormac Taylor
- Kenneth Taylor
- Matthew Taylor
- Michael Taylor
- Nicholas Taylor
- Rowland Taylor
- Hamid Tebyan
- Irmgard Tegeder
- Bin Tean Teh
- Julie Teichroeb
- Miguel Teixeira
- Raphael Teixeira
- Jesus Tejero
- Amalio Telenti
- Carlos Telleria
- Michael Tellier
- Ryan Temel
- Shuqing Teng
- Anders Tengholm
- Vanja Tepavcevic
- Jonathan Terman
- Thomas Terwilliger
- Andrew Teschendorff
- Shannon Tessier
- Charlotte Teunissen
- Charlotte Thålin
- Manuel Théry
- Francis Thackeray
- Thaseem Thajudeen
- Deepshi Thakral
- David Thal
- Dietmar Thal
- Roman Thaler
- Victor Thannickal
- Rudolf Thauer
- Francois‐Xavier Theillet
- Kevin Theis
- Michael Theisen
- Maria Themeli
- Michel Thiebaut de Schotten
- Alexander Thiele
- Meinolf Thiemann
- Cecile Thirant
- Charles Thomas
- François Thomas
- Gary Thomas
- Oliver Thomas
- Walter Thomas
- Barry Thompson
- Linda Thompson
- Michael Thompson
- Nathan Thompson
- Rebecca Thompson
- Jesper Thomsen
- Mads Thomsen
- Rosa Thorolfsdottir
- Simon Thrush
- Chanitra Thuwajit
- Uffe Høgsbro Thygesen
- Lei Tian
- Songhai Tian
- Katharine Tibbetts
- Tibor Tibor
- Ruth Timme
- Sergio Timoteo
- Richard Ting
- Mathieu Tiret
- Amit Tirosh
- Heidi Tissenbaum
- Bjoern Titz
- Zakir Tnimov
- Sokol Todi
- George Tofaris
- Mesut Togacar
- Gaku Tokuda
- Nobuhiko Tokuriki
- Ryutaro Tokutsu
- Iva Tolic
- Vladimir Tolmachev
- Francesco Tomaiuolo
- Katarzyna Tomala
- María Tomas
- Lubomir Tomaska
- Michael Tomasson
- Pablo Tomatis
- Kazunori Tomita
- James Tomkins
- Tatsuya Tomo
- Atsushi Tomokiyo
- Martin Tomov
- Alessandro Tondelli
- Mancy Tong
- Jacek Topczewski
- Timothy Topper
- Colin Torney
- Giusy Tornillo
- Davis Torrejon
- Eduardo Torres
- Marcelo Der Torossian Torres
- Nicole Torres-Tamayo
- Tiina Tosens
- Miklos Toth
- Agnes Toth-Petroczy
- David Toubiana
- Kazushige Touhara
- John Tower
- Shoichi Toyabe
- Ash Toye
- Masanori Toyofuku
- Philip Trackman
- Joseph Tracy
- Marc Tramier
- Gilles Travé
- Mark Travassos
- Michael Traxlmayr
- Stephen Traynelis
- Bryan Traynor
- Adrian Treves
- Juan Luis Trincado Alonso
- Todd Triplett
- Madhukar Trivedi
- Marie-Bérengère Troadec
- Ras Trokovic
- Rodrigo Troncoso
- Matthias Trost
- François Trottein
- Ian Trounce
- Marta Trub
- Emiliano Trucchi
- George Truskey
- Matthias Truttmann
- Aphrodite Tsaballa
- Ageliki Tsagaratou
- Francis Tsai
- Isheng Jason Tsai
- Pei-San Tsai
- Scott Tsai
- Aristidis Tsatsakis
- Eric Tse
- Boo Tseng
- Jack Tseng
- Makoto Tsuda
- Yoshiaki Tsuji
- Yusuke Tsukatani
- Benjamin Tully
- Alice Turdo
- Yatish Turkahia
- Alan Turner
- Christopher Turner
- Beata Turonova
- Timo Tuuri
- William Tyler
- Esa Tyystjärvi
- Alexander Tzagoloff
- Jordan Ubbens
- Eiji Uchibe
- Lucina Uddin
- Severin Uebbing
- Hiroki Ueda
- Emil Uffelmann
- Kathrin Ulrich
- Peter Ulvskov
- Charlie Underwood
- Suraj Unniappan
- David Unwin
- Atul Upadhyay
- Dustin Updike
- Ryuta Uraki
- Iztok Urbančič
- Caitlin Uren
- Jose Utrilla
- Vladimir Uversky
- Brigette Uwimana
- Ahmet Kerim Uysal
- Åshild Vågene
- Ákos Végvári
- Germán Vélez‐Reyes
- Beat Rolf Vögeli
- Gerben Vader
- Bharat Vaidyanathan
- Alexander Vainstein
- Sàndor Vajda
- Tomas Valenta
- Antoni Valero-Cabré
- Sophie Valkenburg
- Eugene Valkov
- Léo Valon
- Angel Valverde
- Oscar Valverde-Barrantes
- Daan van Aalten
- Jim van Belzen
- Sebastian van de Linde
- Rieneke van de Ven-van Balken
- Bastijn van den Boom
- Johan van den Hoogen
- Alexander van der Linden
- Philip van der Merwe
- Jenny van der Wijst
- Karine van Doninck
- Schuyler van Engelenburg
- Hugo Van Ingen
- Jochem van Kempen
- Steven van Lanen
- Christiaan van Ooij
- Mark van Raaij
- Donelly van Schalkwyk
- Bruno van Swinderen
- Sven van Teeffelen
- Herman van Tilbeurgh
- Sandra van Vliet
- Lodewijk van Walraven
- Virginie van Wassenhove
- Gert van Zyl
- Robert Vanburen
- Ivana Vancurova
- Pieter Vanden Berghe
- Alexis Vandenbon
- Barbara Vanderhyden
- Kelli Vandussen
- Jean Vannier
- Dolors Vaqué
- Alejandro Vaquero
- Vitya Vardanyan
- Eugene Varfolomeev
- Zsuzsanna Varga
- Luis Vargas-Chacoff
- David Varricchio
- Ajith Vasanthakumar
- Robert Vassar
- Anna Vavrinova
- Ivone Vaz-Moreira
- Alexei Vazquez
- Andres Vazquez-Torres
- Andrew Veale
- Maurizio Vedani
- Mario Vega
- Libor Velisek
- Jana Veliskova
- Licio Velloso
- Laurent Venance
- Sriram Venneti
- Alessandro Venosa
- Henrietta Venter
- Victor Vergara
- Alexis Verger
- Paul Verkade
- Josh Vermaas
- Geerat Vermeij
- Frederik Verweij
- Tom Vettenburg
- Vaiva Vezys
- Felix Viana
- Maria Vicent
- Mark Vickers
- Mayra Vidal
- Miguel Vidal
- Gianpiero Vigani
- Margarita Vigodner
- Matam Vijay-Kumar
- Ranjit Vijayan
- Sukumar Vijayaraghavan
- Steven Vik
- Olaia Vila
- John Viles
- Bjarni Vilhjalmsson
- Carlos Villalba Galea
- Fernando Villanea
- Juan Carlos Villarreal
- Mark Viney
- Raquel Vinhas
- David Virshup
- Lydia Visser
- Jane Visvader
- David Vocadlo
- Adam Vogel
- Alfried Vogler
- Fabian Voigt
- Vootele Voikar
- Laura Volpicelli-Daley
- Charlotte Von Gall
- Ferdinand Von Meyenn
- Annemarie Voorberg-van der Wel
- Alexey Vorobev
- Peter Voshol
- Daniel Voytas
- Menno Vriens
- Joseph Vu
- Ly Vu
- Ngoc Vu
- Vladyslav Vyazovskiy
- Richard Wade-Martins
- Nicholas Waglechner
- Samuel Wagner
- Rizwan Wahab
- Walter Wahli
- Eric Wait
- Patrik Waldmann
- Maximilian Waldner
- Annemiek Walenkamp
- Daniel Wall
- Kenneth Wallace
- Kyle Walsh
- Julian Walters
- Kylie Walters
- Benjamin Walther
- Dongshi Wan
- Chaolong Wang
- Chi Wang
- Christian Wang
- Dong Wang
- Fengping Wang
- Guangyu Wang
- Guey-Shin Wang
- Hao Wang
- Harris Wang
- Hong Wang
- Hui Wang
- Jianhua Wang
- Jimin Wang
- Jing Wang
- Jianjie Wang
- John Wang
- Juan Wang
- Jun Wang
- Kai Wang
- Kankan Wang
- Kejian Wang
- Li Wang
- Liang Wang
- Lin-Fa Wang
- Lizhong Wang
- Ming-Bo Wang
- Minyan Wang
- Pei-Ling Wang
- Shaobin Wang
- Shaohe Wang
- Shengjun Wang
- Sihong Wang
- Wenqi Wang
- Wei Wang
- Xiaoming Wang
- Xiaoxue Wang
- Xin Wang
- Xinping Wang
- Xu Wang
- Xuefeng Wang
- Yangming Wang
- Yi Wang
- Yi-Ping Wang
- Yong Wang
- Yunbing Wang
- Zefang Wang
- Zhanhui Wang
- Zhao Wang
- Zhao-Qi Wang
- Zhe Wang
- Zhengfei Wang
- Zhenxun Wang
- Zhigang Wang
- Zhihui Wang
- Phurpa Wangchuk
- Carol Ward
- Robert Ward
- Sally Ward
- Hannah Wardill
- Alan Warren
- Chris Warren
- Leonhard Waschke
- Takamitsu Watanabe
- Toru Watanabe
- Robert Waterhouse
- Corey Watson
- Mick Watson
- Adam Wax
- Gary Wayman
- Valerie Weaver
- Ashley Webb
- Kathrin Weber
- Tobias Wech
- Robbee Wedow
- Gunter Wegener
- Jakob Wegener
- Michael Wegner
- Seraphine Wegner
- Gong-Hong Wei
- Guo-Wei Wei
- Guowei Wei
- Kevin Wei
- Kuo-Chen Wei
- Qing Wei
- Wenyi Wei
- Thomas Weichhart
- Ralph Weidner
- Roberto Weigert
- Conrad Weihl
- Cornelis Weijer
- Tanita Wein
- Els Weinans
- Kevin Weiner
- Arne Weinhold
- Gordon Weir
- David Weis
- Sebastian Weis
- Ellen Weisberg
- Lucien Weiss
- Omer Weissbrod
- Matthias Weissensteiner
- Haitao Wen
- Ben Wendel
- Nicole Wenderoth
- Carolin Charlotte Wendling
- Tamra Werbowetski-Ogilvie
- Benjamin Werner
- Jan Wessel
- Niels Wessel
- Michael Wessels
- Daniel Wesson
- Michelle West
- Alexander Westermann
- Benedikt Westermann
- Peter Westley
- Jacqueline Whatmore
- Adam Wheatley
- Glen Wheeler
- John White
- Richard White
- James Whiting
- Dorota Wianowska
- Carolin Wichmann
- Anja Wiechmann
- Bertram Wiedenmann
- Brian Wiegmann
- Timothy Wiggin
- Craig Wilen
- Luke Wiley
- Steven Wilhelm
- Thomas Wilhelm
- André Barretto Bruno Wilke
- Jeremy Wilkinson
- Rob Wilkinson
- Frances Williams
- Jesse Williams
- Larissa Williams
- Tom Williams
- David Williams, Jr.
- Gerald Willimsky
- Monte Willis
- Ben Willmore
- Karin Willquist
- Ingo Willuhn
- Donald Wilson
- Gregory Wilson
- Justin Wilson
- Laurence Wilson
- Robert Wilson
- Thomas Wilson
- William Wilson
- Patrick Wintrode
- Alison Wirshing
- Aaron Wirsing
- William Wisden
- Robert Wisse
- Laurens Witter
- Cristian Woelk
- Stephanie Wohlgemuth
- Elia Tait Wojno
- Francis Wolenski
- Eva Wolf
- Joanna Wolfe
- Matthew Wolfgang
- Mariana Wolfner
- Isabelle Wolowczuk
- Knut Woltjen
- Ten-Tsao Wong
- Terence Wong
- Tania Wong Fok Lung
- Dori Woods
- Jeremy Woodward
- Robyn Wootton
- Thierry Work
- Thomas Worzfeld
- Gerry Wright
- Chenggang Wu
- Guojun Wu
- Hao Wu
- Hongju Wu
- Jiahe Wu
- Jianqiang Wu
- Jie Wu
- Long-Jun Wu
- Ronghu Wu
- Shuang Wu
- Xianghao Wu
- Xiaohua Wu
- Yang Wu
- Zhaohui Wu
- Elsio Wunder
- Gerhard Wunderlich
- Michael Wyatt
- Doug Wylie
- Ursula Wyneken
- Tony Wyss-Coray
- Peng Xi
- Rongwen Xi
- Zhiyong Xi
- Tian Xia
- Xiao-Jian Xia
- Yang Xia
- Yin Xia
- Yuqiong Xia
- Shengnan Xiang
- Yangfei Xiang
- Ye Xiang
- Bailong Xiao
- Guozhi Xiao
- Junyu Xiao
- Shuhai Xiao
- Linglin Xie
- Wei Xie
- Da Xing
- Deyin Xing
- Jinchuan Xing
- Junji Xing
- Yaowu Xing
- Bo Xiong
- Yong Xiong
- Chi Xu
- Fei Xu
- Heping Xu
- Jianming Xu
- Jie Xu
- Lin Xu
- Mike Xu
- Peng Xu
- Wenging Xu
- Xiaoji Xu
- Yong Xu
- Youwei Xu
- Gaya Yadav
- Hiroshi Yagi
- Ryosuke Yamada
- Yoshiki Yamaguchi
- Masayuki Yamamoto
- Akihiro Yamanaka
- Hidenori Yamasue
- Qingpi Yan
- Zhengbing Yan
- Itai Yanai
- Sumino Yanase
- Annan Yang
- Bing Yang
- Bo Yang
- Chao-Chun Yang
- Fentang Yang
- Hao Yang
- Jinsung Yang
- Pengyi Yang
- Shuang Yang
- Wanling Yang
- Wei Yang
- Weijian Yang
- Xiaojing Yang
- Xiaojun Yang
- Yang Yang
- Zhenyan Yang
- Mark Yarchoan
- Robert Yarchoan
- Venkateswarlu Yarlagadda
- Hussein Yassine
- John Yates
- Michelle Yau
- Azam Yazdani
- Bang-Ce Ye
- Dan Ye
- Jianping Ye
- Joseph Yeeles
- Kemal Yelekçi
- Alexei Yeliseev
- Loïc Yengo
- Leslie Yeo
- Ali Ekrem Yesilkanal
- Jonathan Yewdell
- Won Cheol Yim
- Dean Yimlamai
- Xiling Yin
- Yeshi Yin
- Kwok-Ho Yip
- Joo-Yeon Yoo
- Gyeong Mee Yoon
- Hwan Su Yoon
- Yuji Yoshiko
- Shige Yoshimura
- Tadayuki Yoshitake
- Chenjiang You
- Sixian You
- Arabella Young
- Eric Young
- Kendra Young
- Paul Young
- Pierce Young
- Ryland Young
- Walter Young
- Neil Youngson
- Ihab Younis
- Noha Yousri
- Inmaculada Yruela
- Chunmei Yu
- Edward Yu
- Haibo Yu
- Han-Gang Yu
- Hongwei Yu
- Jianing Yu
- Jin Yu
- Kebing Yu
- Ri-Qing Yu
- Shan Ping Yu
- Xiuping Yu
- Xuekui Yu
- Zhengquan Yu
- Zhongbo Yu
- Zuoren Yu
- Guangyin Yuan
- Lester Yuan
- Peng Yuan
- Shuguang Yuan
- Yao-Wu Yuan
- Min Yue
- Iwata Yuji
- Peter Yunker
- Joseph Zabner
- Stephanie Zaffran
- Joseph Zaia
- Ran Zalk
- Tomaso Zambelli
- Elias Zambidis
- Laura Zannini
- Giuseppe Zanotti
- Theodore Zanto
- Juan Zapata
- Ryan Zapotocny
- Roberta Zappasodi
- Laure Zaragosi
- Kenneth Zaret
- Natalia Zaretskaya
- Assaf Zaritsky
- Michael Zavada
- Anton Zavialov
- Mark Zaydman
- Lynn Zechiedrich
- Jürgen Zeier
- Aleksej Zelezniak
- Oana Zeleznik
- Jian Zeng
- Ping Zeng
- Zixian Zeng
- Tsungai Zengeya
- Karsten Zengler
- Gabriel Zentner
- Valerio Zerbi
- Helen Zgurskaya
- Weiwei Zhai
- Kateryna Zhalnina
- Lixing Zhan
- Wenbo Zhan
- Bin Zhang
- Chao Zhang
- Chiyu Zhang
- Denghai Zhang
- Di Zhang
- Faming Zhang
- Fan Zhang
- Feng Zhang
- Gong Zhang
- Heng Zhang
- Hongkai Zhang
- Huijie Zhang
- Huiliang Zhang
- Jian Zhang
- Jianxiang Zhang
- Jianzhi Zhang
- Jin Zhang
- Jiwei Zhang
- Kaihua Zhang
- Kaiming Zhang
- Le Zhang
- Lei Zhang
- Lin Zhang
- Ling-Juan Zhang
- Linyi Zhang
- Liqun Zhang
- Lisha Zhang
- Liwen Zhang
- Lu Zhang
- Mary Zhang
- Peijing Zhang
- Qiangfeng Zhang
- Qinghua Zhang
- Qingzhu Zhang
- Ru Zhang
- Rui Zhang
- Wenjing Zhang
- Xiaodong Zhang
- Xiaohui Zhang
- Xiaowei Zhang
- Xiaoxue Zhang
- Xiaoyang Zhang
- Xing-Hua Zhang
- Xingtan Zhang
- Xinjun Zhang
- Xuewu Zhang
- Xueyong Zhang
- Yijing Zhang
- Zhi Zhang
- Zhihao Zhang
- Bo Zhao
- Chen Zhao
- Chunzhao Zhao
- Gongpu Zhao
- Hien Tran Zhao
- Hu Zhao
- Huabin Zhao
- Huaying Zhao
- Huiying Zhao
- Jian Zhao
- Jianjun Zhao
- Lei Zhao
- Weian Zhao
- Xiaolan Zhao
- Xing-Ming Zhao
- Yanxiang Zhao
- Yun Zhao
- Yunde Zhao
- Zhen Zhao
- Gao Zhaobing
- Danping Zheng
- Jie Zheng
- Wenyi Zheng
- Xiuwen Zheng
- Y. George Zheng
- Yuebing Zheng
- Guocai Zhong
- Chengji Zhou
- Haibo Zhou
- Joe Zhou
- Ming-Ming Zhou
- Qingxuan Zhou
- Zhemin Zhou
- Zhong-Wei Zhou
- Zhuo Zhou
- Fu-Yuan Zhu
- Hongliang Zhu
- Hua Zhu
- Le-Le Zhu
- Lifeng Zhu
- Maoyan Zhu
- Wenhan Zhu
- Xiaohui Zhu
- Zheng-Jiang Zhu
- Shougang Zhuang
- Victor Zhurkin
- Josef Zicha
- Wilma Ziebuhr
- Amanda Ziegler
- Stefan Zielonka
- Tomáš Zikmund
- Danila Zimenkov
- Manuel Zimmer
- Geraldine Zimmer-Bensch
- Christine Zimmermann
- Wolfram Zimmermann
- Lucie Zinger
- Sandra Zinkel
- Annelies Zinkernagel
- Natalia Zinovieva
- Warren Zipfel
- Didier Zoccola
- Janeta Zoldan
- Marianna Zolotovskaia
- Katerina Zorina-Lichtenwalter
- Antonio Zorzano
- Jitao Zou
- Qiang Zou
- Lejla Zubcevic
- Andrea Zuccolo
- Daming Zuo
- Christian Zuppinger
- Ipshita Zutshi
- Paul Zwack
- Arthur Zwaenepoel
- Martijn Zwama
- An Zwijsen
- Jan Zylicz

